# An optimized fluorescent reporter enables rapid and cost-effective quantification of regulated secretion from neuroendocrine cells

**DOI:** 10.3389/fendo.2025.1640601

**Published:** 2025-08-18

**Authors:** Theodore Carter, Alice McTavish, Cedric S. Asensio

**Affiliations:** Department of Biological Sciences, University of Denver, Denver, CO, United States

**Keywords:** insulin, regulated secretion, beta cells, fluorescent reporter, PC12 Cells, INS-1 cells, TIRF, plate reader

## Abstract

The ability to quantify protein secretion is critical for studying the secretory pathway. This is particularly important in endocrine cells where dysregulated hormone secretion is associated with the development of diseases such as type 2 diabetes. To measure protein secretion, researchers have previously relied on techniques such as ELISA, RIA and Western blot, which all present limitations, including cost and time consumption. To address these challenges, we developed a plate reader-based assay using an optimized red fluorescent reporter, NPY-sfCherry3c. This reporter showed enhanced expression, proper sorting into secretory granules, and robust secretion from both INS-1 832/13 and PC12 cells. As NPY-sfCherry3c displayed better signal-to-background ratio compared to previously published reporters (e.g. NPY-GFP, NPY-mCherry), secretion could easily be detected within a few minutes of stimulation, demonstrating the assay’s enhanced sensitivity. Our results suggest that NPY-sfCherry3c is a valuable tool to perform rapid and cost-effective secretion assays from neuroendocrine cells.

## Introduction

The regulated secretion of proteins is associated with many physiological processes, but plays a central role in endocrinology, where the release of peptide hormones such as insulin and glucagon are essential for glucose homeostasis. Regulated secretion involves specialized vesicles called secretory granules (SGs) or large dense-core vesicles, which facilitate the intracellular storage and mediate the release of these peptide hormones. In contrast to the constitutive secretory pathway, which continuously releases newly synthesized secretory proteins, regulated secretion is triggered by specific stimuli that lead to SG exocytosis.

Tools that allow for the quantification of protein secretion are critical to study how cells respond to stimuli, how certain proteins may modulate the process, or to test the effect of small molecules. Traditionally, a variety of methods have been used to quantify protein secretion, including Enzyme-Linked Immunosorbent Assays (ELISAs), Radioimmunoassays (RIAs) and Western blots (1–3). Although ELISAs and RIAs effectively quantify secreted proteins such as insulin, they are often costly, time-consuming and prone to error due to a multitude of handling steps. There are also drawbacks to performing Western blots, particularly in the case of low-abundance and low molecular weight processed peptides. For example, insulin is only 6 kDa and thus cannot easily be detected on standard acrylamide gels. This problem is further amplified when only a small fraction of the cellular content is released under stimulatory conditions, making the routine analysis of neuroendocrine cell secretion more complicated.

To address these challenges, we aimed to develop a faster and cost-effective assay that utilizes a fluorescent tagged reporter and a plate reader as an alternative to traditional methods. Although insulin would be an ideal secretion marker for beta cells, tagging it with fluorescent proteins has historically been difficult (4). It can be achieved by introducing the fluorescent reporter within the C-peptide, which is cleaved by prohormone convertase enzymes during granule maturation (5). The fluorescently tagged C-peptide remains in the granule with mature insulin and can be released upon stimulation. However, tagging insulin with fluorescent proteins presents limitations, including the potential to induce significant ER stress. Although the more recent development of superfolder GFP (sfGFP), which exhibits improved folding kinetics while maintaining fluorescence (6), has further enhanced the tagging of insulin (7), insulin is not endogenously expressed by many endocrine cells, making it less ideal as a universal marker across different cell types. As a result, we opted for an alternative marker with broader expression across endocrine cells - neuropeptide Y (NPY) (8–10)- which has been successfully tagged before (11) and used extensively as a reporter (12–14).

Here we describe the development and validation of NPY-sfCherry3c as a fluorescent reporter optimized for use in a cost-effective, plate-reader-based secretion assay. NPY-sfCherry3c exhibits robust expression and appropriate sorting in both INS-1 832/13 and PC12 cells, enabling sensitive detection of basal and stimulated secretion with an improved signal-to-background ratio. In addition to its utility in plate-reader assays, the reporter is also well-suited for live-cell imaging using total internal reflection fluorescence (TIRF) microscopy. We anticipate that this reporter, and the accompanying assays, will provide a valuable and accessible tool for researchers studying neuroendocrine regulated secretion.

## Results

We previously developed a plate-reader based assay to quantify regulated secretion from rat pheochromocytoma PC12 cells using the SG marker Atrial Natriuretic Factor (ANF) fused to emeraldGFP (emdGFP) (15, 16). Although ANF can be expressed in PC12 cells, it is not endogenously expressed by most neuroendocrine cells. Among other potential SG reporters, the soluble cargo NPY is an interesting candidate as it is found in SGs of both PC12 and INS-1 832/13 cells, along with most other (neuro-)endocrine cell lines. As many of the constructs we use in our research are already tagged with GFP, we decided to develop a red fluorescent NPY reporter. One of the key benefits to using a red fluorescent reporter over a green fluorescent reporter is the ability to perform multi-color experiments, allowing for the observation of more than one protein at a time, while avoiding spectral overlap (17, 18). Furthermore, red fluorescent proteins (RFPs) have reduced autofluorescence compared to GFPs allowing for greater signal-to-background ratios, resulting in cleaner and more accurate data (19). An additional advantage of RFPs is their relatively low degree of phototoxicity in cells (20).

### NPY-sfCherry reporters exhibit enhanced expression and proper sorting in insulinoma cells

**Figure 1:**
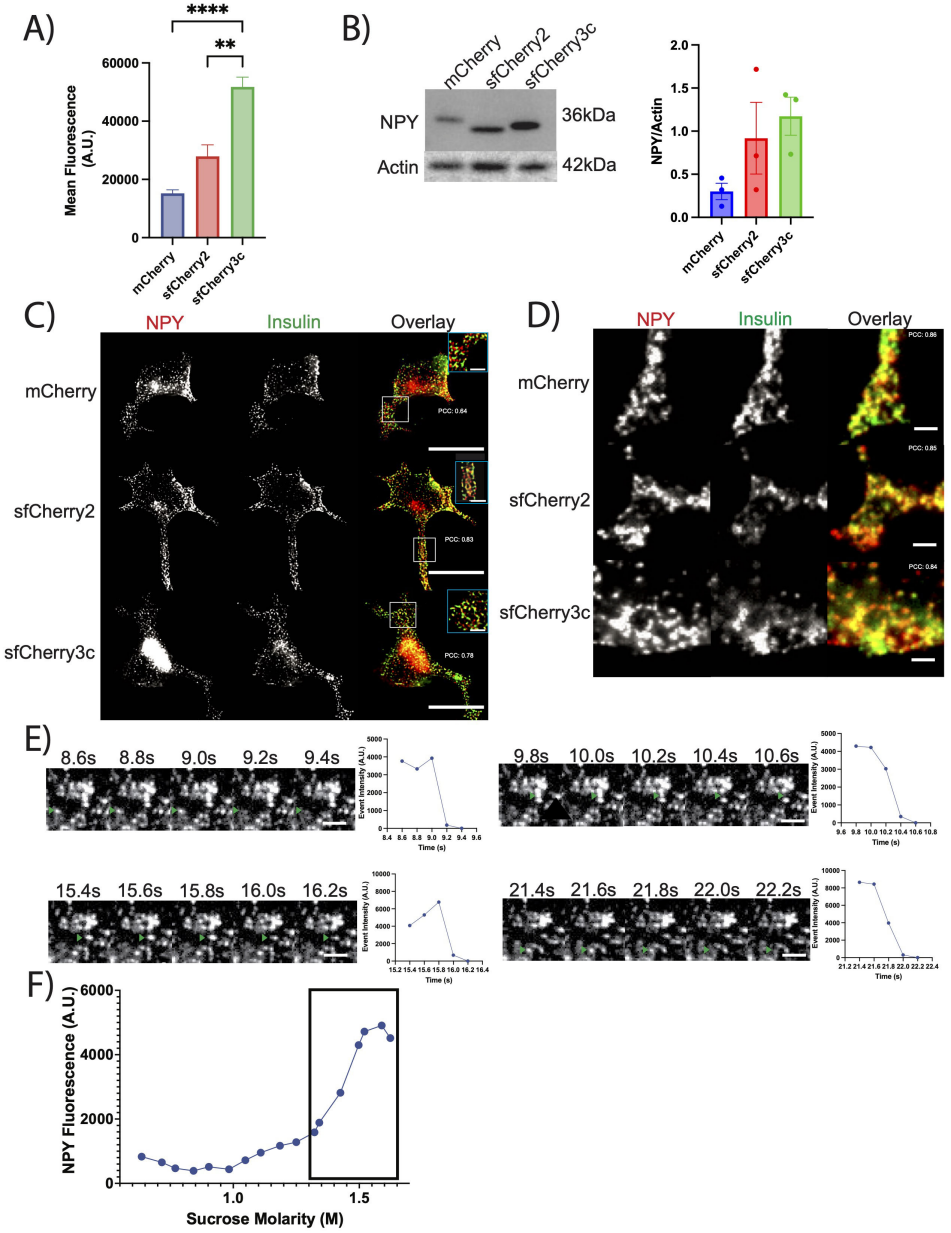
**(A)** INS-1 832/13 cells transiently transfected with the indicated reporters were analyzed by flow cytometry. Data indicate the mean fluorescence. **p<0.01, ****p<0.0001 by one-way ANOVA (>10,000 cells). The bar graphs indicate mean values. **(B)** INS-1 832/13 cells transiently transfected with the indicated reporter were immunoblotted for NPY. The bar graphs indicate mean values of NPY normalized to Actin (n = 3 independent transfections). Data not statistically significant by one-way ANOVA. **(C, D)** INS-1 832/13 cells were transiently transfected with the indicated reporter (red), immunostained for insulin (green) and imaged using widefield fluorescence microscopy **(C)** and TIRF microscopy **(D)**. Scale bar indicates 20µm, while inset scale bar indicates 5µm for widefield and 2µm for TIRF. Pearson’s correlation coefficient indicated on respective images. **(E)** Representative examples of NPY-sfCherry3c-positive exocytotic events captured by live TIRF-microscopy. Images were taken every 200ms. Plot on the right indicates event intensity at the given time points. 40mM KCl added at time=0s. Scale bar indicates 2µm. **(F)** INS-1 832/13 cells transiently transfected with NPY-sfCherry3c and fractionated based on density. Secretory granule fractions indicated by box around data points.

Recently developed sfCherry proteins (sfCherry, sfCherry2, and sfCherry3c) offer improved folding and brightness compared to mCherry making them an attractive choice (21, 22). We designed both NPY-sfCherry2 and NPY-sfCherry3c for comparison with previously developed NPY-mCherry. We first compared the signal intensity of NPY-mCherry, NPY-sfCherry2, and NPY-sfCherry3c in transfected INS-1 832/13 cells using flow cytometry. Cells expressing NPY-sfCherry3c showed significantly stronger mean fluorescence than cells expressing NPY-mCherry or NPY-sfCherry2 (**Figure 1A**, **Supplementary Figure 1**). Western blot analysis of cell lysates using an antibody against NPY showed increased, albeit not statistically significant, expression of NPY-sfCherry2 and NPY-sfCherry3c compared to NPY-mCherry (**Figure 1B**). These results suggest that the superfolder constructs likely express better due to improved folding, and that NPY-sfCherry3c exhibits enhanced fluorescence. We then immunostained cells transfected with the various NPY reporters alongside insulin to assess their sorting into SGs (**Figure 1C**). We observed significant overlap between NPY and insulin signals by widefield fluorescence microscopy, indicating that all reporters correctly localized to SGs, supported by a high Pearson’s correlation coefficient (NPY-mCherry: 0.64, NPY-sfCherry2: 0.83, NPY-sfCherry3c: 0.78). To further confirm these findings, we imaged the transfected cells using TIRF microscopy and found that all three NPY reporters showed high overlap with insulin, also supported by a high Pearson’s correlation coefficient (NPY-mCherry: 0.86, NPY-sfCherry2: 0.85, NPY-sfCherry3c: 0.84), further supporting the proper localization of the reporters (**Figure 1D**). Previously, mCherry has been documented to form aggregates in hippocampal neurons (23), but we observed no such aggregates using NPY-sfCherry3c under our experimental conditions. We also performed live-imaging of NPY-sfCherry3c transfected INS-1 832/13 cells using TIRF and could observe NPY-sfCherry3c exocytotic events in response to depolarization with 40mM KCl, indicating that NPY-sfCherry3c properly sorts into functional granules (**Figure 1E**). Finally, we used equilibrium sedimentation through sucrose to determine the distribution of NPY-sfCherry3c from transfected INS-1 832/13 cells (**Figure 1F**). The reporter accumulated primarily in fractions corresponding to 1.4-1.6M sucrose, which we previously identified as insulin-positive by ELISA (24).

### NPY-sfCherry3c works as a reporter for regulated secretion from insulinoma cells

**Figure 2:**
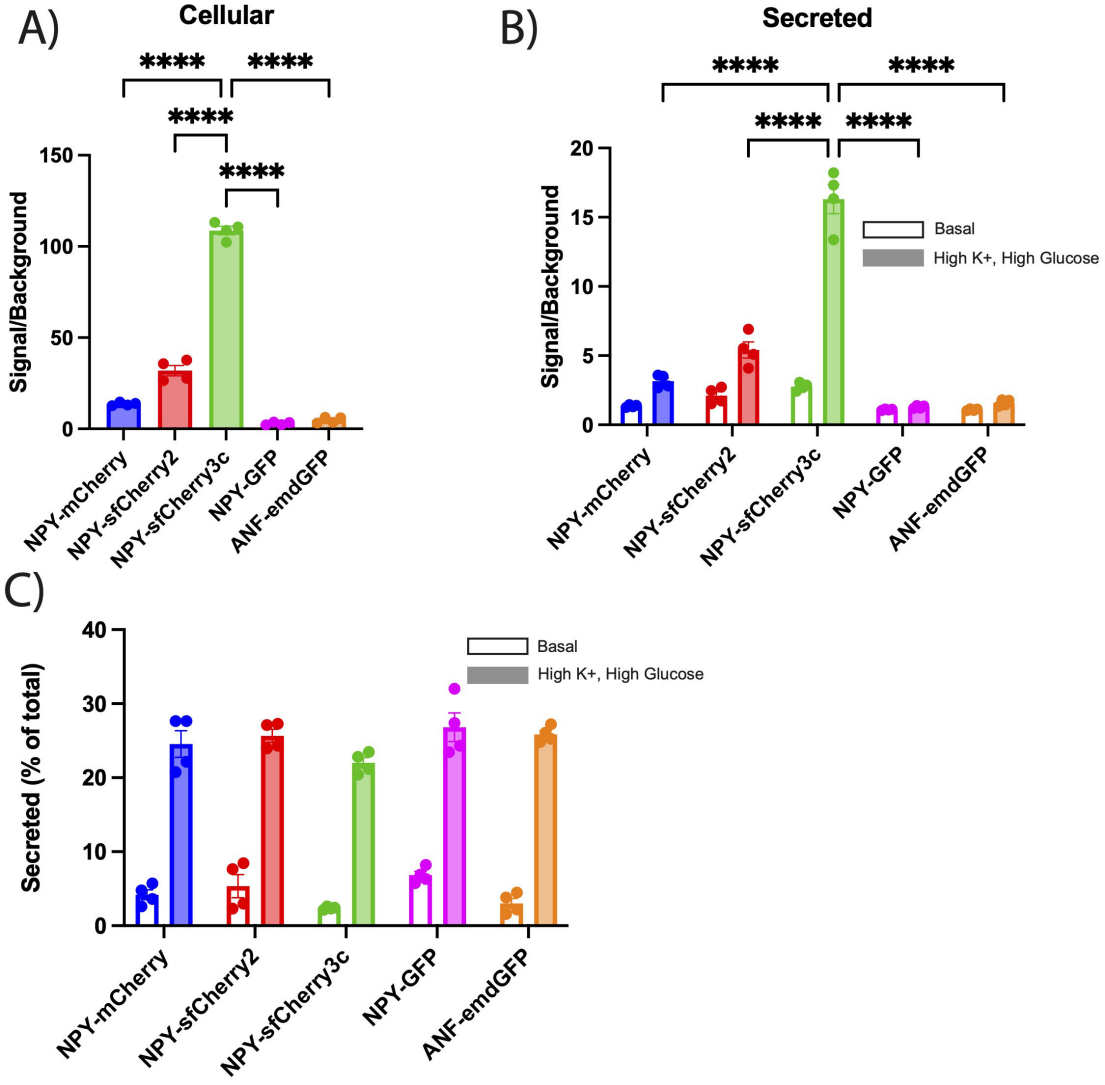
**(A-C)** INS-1 832/13 cells were transiently transfected with the indicated reporters, **(B, C)** washed, and incubated with basal or high K^+^ plus high glucose KRB solution for 2 hours. Fluorescence from cellular and secreted fractions were measured using a plate reader. **(A, B)** The bar graphs show the signal to background ratios of cellular **(A)** or secreted **(B)** fractions for each indicated reporter. Background was determined by measuring fluorescence of untransfected cell lysates and secreted fractions. The bar graphs indicate mean ± s.e.m. Open bars indicate basal secretion, filled bars indicate high K^+^ plus high glucose secretion (n = 4 biological replicates). ****p<0.0001 relative to NPY-sfCherry3c by two-way ANOVA or one-way ANOVA for cellular content. **(C)** Basal and high K^+^ plus high glucose secretion was expressed as a percent of total content. The bar graphs indicate mean ± s.e.m. Open bars indicate basal secretion, filled bars indicate stimulated secretion (n = 4 biological replicates). No statistical significance was observed by two-way ANOVA.

We next tested the performance of the new red fluorescent reporters (NPY-sfCherry2 and NPY-sfCherry3c) in comparison to NPY-mCherry as well as NPY-GFP and ANF-emdGFP, using a secretion assay. For this, INS-1 832/13 cells transfected with the various reporters were lysed and had their fluorescence measured on a plate reader. NPY-sfCherry3c cellular content consistently showed a >100-fold signal-to-background ratio, which is significantly higher than all the other reporters (**Figure 2A**). We then incubated INS-1 832/13 cells transfected with the various reporters for two hours under either basal (KRB) or stimulatory (High K^+^ and High glucose KRB) conditions. After incubation, we measured fluorescence in the secreted fractions using a plate-reader. We observed a similar trend in the secreted fractions, where NPY-sfCherry3c outperformed the other reporters (**Figure 2B**).

When we expressed secretion as a percentage of total cellular fluorescence to account for differences in transfection and expression between reporters, we found that regulated secretion was comparable across all reporters with no statistically significant difference observed (**Figure 2C**). However, NPY-GFP, NPY-mCherry, and NPY-sfCherry2 exhibited higher levels of basal secretion compared to ANF-emdGFP, and NPY-sfCherry3c. We speculate that the increased basal secretion might be due to fluorescence values being too close to background for NPY-mCherry, NPY-sfCherry2 and NPY-GFP. Additionally, we noted that the autofluorescence signal was much stronger for GFP than for Cherry under our experimental conditions and with our plate reader. Due to its enhanced signal, we selected NPY-sfCherry3c as the preferred reporter for further experiments, especially under conditions where secretion may be reduced.

### Detection of glucose stimulated secretion using NPY-sfCherry3c

**Figure 3:**
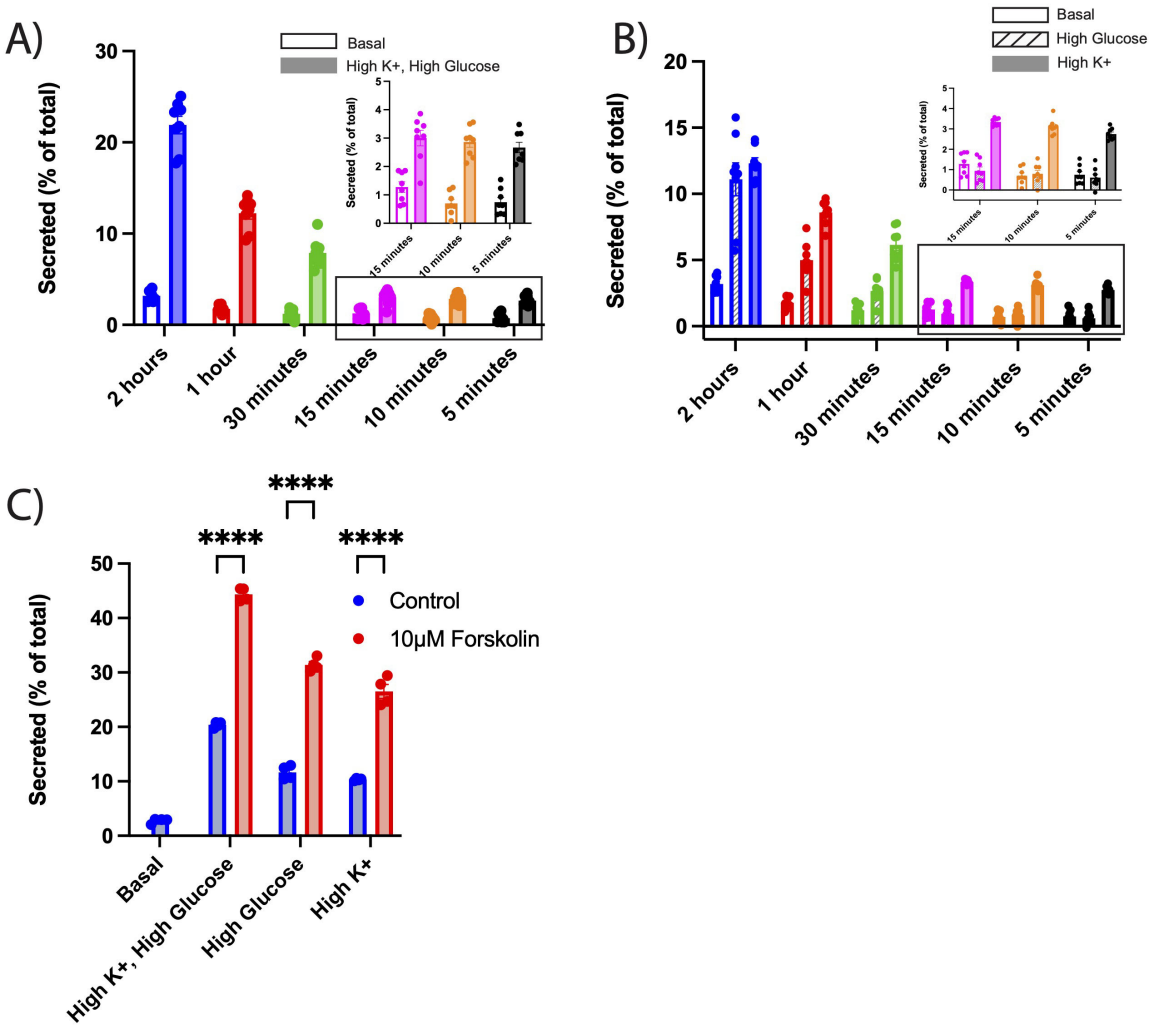
**(A-C)** INS-1 832/13 cells were transiently transfected with NPY-sfCherry3c. Secretion assays were performed as described in **Figure 2**. Cells were incubated either with basal or high K^+^ plus high glucose **(A)**, high glucose **(B)**, or high K^+^ KRB solutions **(B)** with incubation time ranging from 2 hours to 5 minutes. **(A)** Open bars indicate basal secretion, filled bars indicate high K^+^ plus high glucose secretion (n = 8 biological replicates). Data within the rectangle box are shown in the inset for better visualization of individual data points. **(B)** Open bars indicate basal secretion, slashed bars indicate high glucose, filled bars indicates high K+ (n = 8 biological replicates). The bar graphs indicate mean ± s.e.m. Data within the rectangle box are shown in the inset for better visualization of individual data points. **(A, B)** Two-way ANOVA was performed for statistical significance. For clarity of graphs, significance points have been omitted (See **Supplementary Figure 2** for significance points). **(C)** Alternatively, cells were treated with 10μM forskolin and secretion stimulated using high glucose plus high K^+^, high glucose, or high K^+^ KRB solutions for 2 hours. Blue bars indicate untreated secretion, red bars indicate secretion under forskolin. The bar graphs indicate mean ± s.e.m. ****p<0.0001 relative to each condition by two-way ANOVA.

To further evaluate the sensitivity of NPY-sfCherry3c, we tested whether we could detect secretion over shorter intervals (from 2 hours down to 5 minutes) under both basal and stimulatory conditions. Remarkably, we could detect NPY-sfCherry3c secretion after just 5 minutes, demonstrating that this assay with this new reporter is sensitive enough to capture rapid secretion events (**Figure 3A**). Given that INS-1 832/13 cells exhibit glucose-mediated insulin secretion (25), we next measured NPY-sfCherry3c secretion in response to high glucose alone vs depolarization (high K+) alone. Between 5 and 15 minutes, we observed no significant response to glucose alone, while depolarization elicited a secretion response comparable to the combined effects of depolarization and high glucose. This suggests that depolarization primarily drives secretion during the early phase (**Figure 3B**). However, between 30 minutes and 2 hours, the cells showed an increasing response to glucose (**Figure 3B**), indicating a shift towards glucose-mediated secretion over time. These results indicate that our assay can effectively capture both rapid and sustained secretion of secretory cargo under various stimulatory conditions with high accuracy and reproducibility.

### Forskolin potentiates glucose and depolarization mediated NPY-sfCherry3c secretion

We next tested whether this assay would detect the effects of established potentiators of insulin secretion. Forskolin, which elevate cAMP levels in cells, enhances insulin secretion in both animal and cell models (26). We examined NPY-sfCherry3c secretion in INS-1 832/13 cells in the presence and absence of forskolin. Under all stimulatory secretion conditions (high glucose, depolarization, high glucose + depolarization), we observed a significant increase in NPY-sfCherry3c secretion (**Figure 3C**), demonstrating this assay’s ability to detect changes in secretion in response to a potentiator.

### PC12 cells display robust secretion of NPY-sfCherry3c

**Figure 4:**
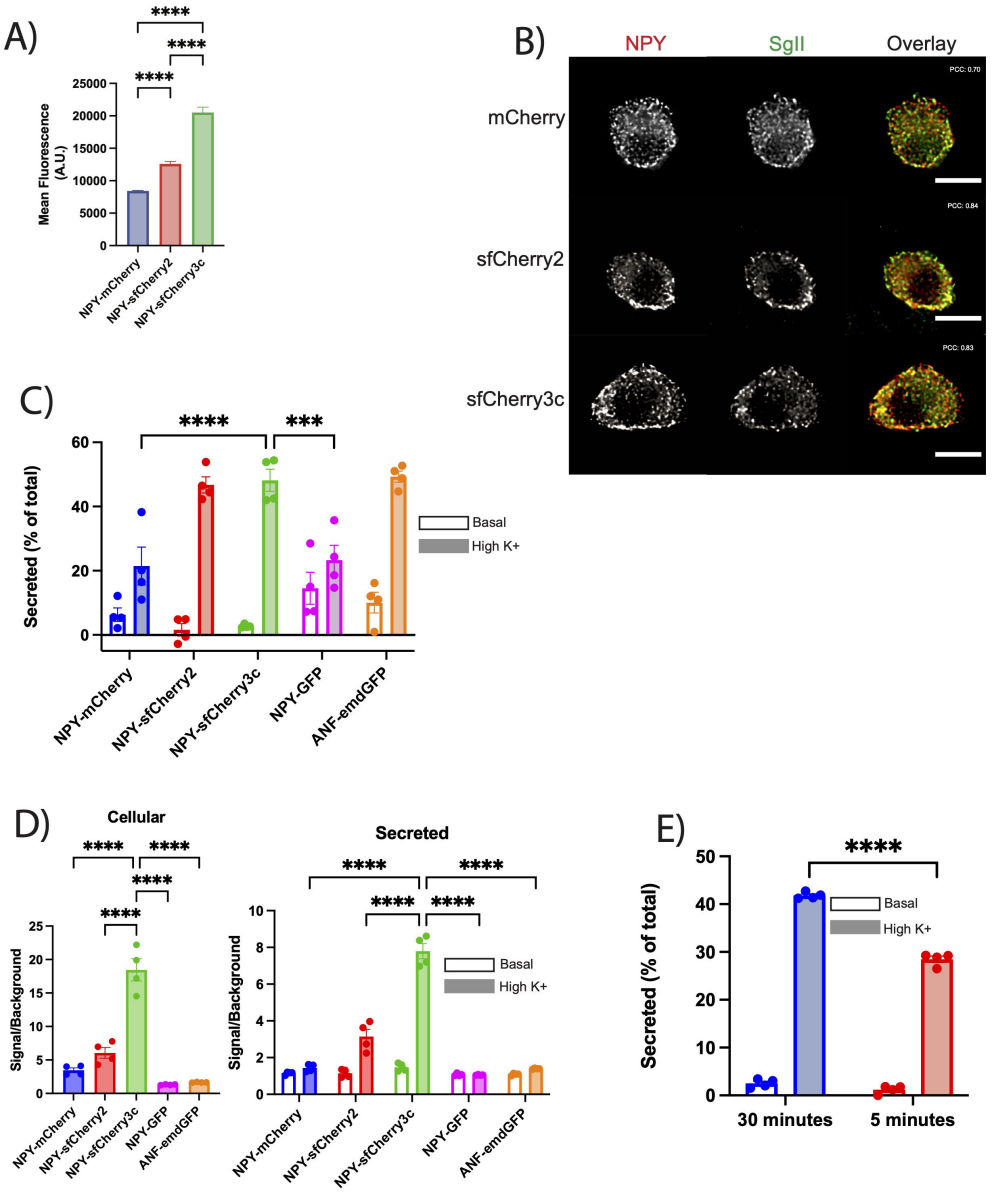
**(A)** PC12 cells transiently transfected with the indicated reporters were analyzed by flow cytometry. Data indicate the mean fluorescence. The bar graphs indicate mean ± s.e.m. ****p<0.0001 by one-way ANOVA (>10,000 cells). **(B)** PC12 cells were transiently transfected with the indicated reporter (red), immunostained for SgII (green) and imaged using widefield fluorescence microscopy. Scale bar indicates 10µm. Pearson’s correlation coefficient indicated on respective images. **(C)** PC12 cells were transiently transfected with the indicated reporters, washed, and incubated with basal or stimulated (high K^+^) Tyrode solution for 30-minute. Fluorescence from cellular and secreted fractions were measured using a plate reader as described in **Figure 1**. Basal and high K^+^ secretion was expressed as a percent of total content. Open bars indicate basal secretion, filled bars indicate high K^+^ secretion (n = 4 biological replicates). The bar graphs indicate mean ± s.e.m. ***p<0.001, ****p<0.0001 relative to NPY-sfCherry3c by two-way ANOVA. **(D)** The bar graphs show the signal to background ratios of cellular (left) or secreted (right) fractions for each indicated reporter. Background was determined by lysing untransfected cells or collecting supernatant of untransfected cells and measuring fluorescence on plate reader. Open bars indicate basal secretion, filled bars indicate high K^+^ secretion (n = 4 biological replicates). The bar graphs indicate mean ± s.e.m. ****p<0.0001 relative to NPY-sfCherry3c by one-way ANOVA for cellular or two-way ANOVA for secreted. **(E)** The bar graphs show normalized secretion of NPY-sfCherry3c from PC12 cells after 5- and 30-minute incubation. Open bars indicate basal secretion, filled bars indicate high K^+^ secretion (n = 4 biological replicates). The bar graphs indicate mean ± s.e.m. ****p<0.0001 relative to NPY-sfCherry3c by two-way ANOVA.

Having demonstrated the assay’s capability in detecting secretion from INS-1 832/13 cells, we aimed to determine its applicability to other neuroendocrine cells. We transfected PC12 cells with NPY-mCherry, NPY-sfCherry2, and NPY-sfCherry3c, measuring the mean fluorescence of each reporter via flow cytometry as described previously. Similar to INS-1 832/13 cells, PC12 cells expressing NPY-sfCherry3c exhibited increased mean fluorescence (**Figure 4A**, **Supplementary Figure 3**). Next, we immunostained PC12 cells transfected with each reporter alongside the SG marker Secretogranin II (SgII) (**Figure 4B**). All reporters showed overlap with SgII, suggesting proper sorting into SGs, supported by high Pearson’s correlation coefficient (NPY-mCherry: 0.7, NPY-sfCherry2: 0.84, NPY-sfCherry3c: 0.83). To evaluate secretion, we transfected PC12 cells with ANF-emdGFP or the various NPY reporters and performed a secretion assay for 30 minutes under basal or stimulatory (high K+) conditions. We measured cellular and secreted reporters using a plate reader as described previously. ANF-emdGFP, NPY-sfCherry2, and NPY-sfCherry3c displayed similar levels of stimulated secretion, while NPY-GFP and NPY-mCherry showed significantly reduced secretion levels (**Figure 4C**). Additionally, we observed that basal secretion was markedly higher for GFP reporters compared to the two sfCherry reporters. As observed in INS-1 832/13 cells, NPY-sfCherry3c exhibited significantly improved signal-to-background ratio in comparison to the other reporters (**Figure 4D**). Finally, we compared NPY-sfCherry3c secretion after 5 minutes and 30 minutes of stimulation and found that over half of total secretion occurred within the first five minutes (**Figure 4E**), demonstrating the assay’s sensitivity. Altogether these findings indicate that NPY-sfCherry3c serves as an effective, universal reporter for plate-reader-based secretion assays in neuroendocrine cells.

## Discussion

In this study, we developed a sensitive and cost-effective alternative method to ELISA or Western blot for secretion assays using NPY-sfCherry3c, with a rapid turnaround time. Our data show that NPY-sfCherry3c sorts properly to secretory granules and expresses at higher levels than NPY-mCherry, resulting in a high signal to background ratio. We demonstrated that this assay reliably detects secretion within just 5 minutes and distinguishes between different stimuli. We also validated its adaptability by applying it to PC12 cells with minimal adjustments. Although it was not explored in this study, we also acknowledge a broader applicability for NPY-sfCherry3c outside of secretion assays, for the use of multi-color imaging of green- and red-fluorescent reporters. For example, NPY-sfCherry3c could be further utilized in TIRF by co-transfecting with NPY-pHluorin, using NPY-sfCherry3c to identify expressing cells and to observe docked granule behavior, while NPY-pHluorin can supplement by easily identifying exocytotic events. While we believe that this assay will be easily adaptable to other neuroendocrine cell lines, we cannot guarantee its applicability due to our testing only being performed in INS-1 832/13 and PC12 cells.

Compared to ELISA, our NPY-sfCherry3c secretion assay offers substantial cost-savings. A single 96-well ELISA plate costs approximately $680. After accounting for 7 standards and a blank in triplicates (24 wells), only 72 wells remain for samples. Including both secreted and corresponding lysates further limits the capacity to 36 samples per plate. In contrast, our assay primarily requires transfection reagents and a 96-well black plate. Runing 36 samples costs around $50, over 12 times less than an ELISA (**Supplementary Table S2**). In addition, unlike ELISA plates, which are single-use and incur the same cost regardless of sample number, our assay allows to reuse any of the remaining wells on the same plate. With its speed, scalability, and affordability, the NPY-sfCherry3c assay provides a compelling alternative to traditional secretion assays. In the context of a challenging funding climate, adopting cost-efficient approaches like this assay is increasingly important for maximizing research output while staying within limited budgets.

## Materials and methods

### Cell culture

INS-1 832/13 cells were originally received as a gift from Dr. Ailion at the University of Washington. INS-1 832/13 cells were cultured in RPMI 1640 medium (GenClone, 25-506), supplemented with 10% FBS (GenClone, 25-525), 1mM Sodium Pyruvate (Cytiva, SH30239.01), 10mM HEPES (GoldBio, H-400-1), and 50µM 2-Mercaptoethanol (Sigma, M3148-25ML). For 5mM and 1.5mM glucose reset, INS-1 832/13 cells were cultured in RPMI 1640 without glucose (USBiological, R8999-13), supplemented with either 5mM or 1.5mM glucose (Sigma, G8769-100ML), along with 10% FBS, 1mM sodium pyruvate, and 10mM HEPES. PC12 cells were originally received as a gift from Dr. Kelly at UCSF. PC12 cells were cultured in DMEM (GenClone, 25-501), supplemented with 10% Horse Serum (Gibco, 16050-122), and 5% Cosmic Calf Serum (HyClone, SH30087.03). Cells were routinely tested for the absence of mycoplasma using a detection kit (ABM, G238).

### Cloning

All gene blocks were purchased from Twist Bioscience and assembled using Gibson Assembly Master Mix (NEB, E2611S). NPY-mCherry, NPY-sfCherry2, and NPY-sfCherry3c were assembled using pCAGGS as the backbone (**Supplementary Table S1**). Following assembly, plasmids were transformed into home-made DH5α competent cells and then grown on agar plates with Carbenicilin (GoldBio, C-103-25) for pCAGGS. Individual colonies were picked and inoculated for 16 hours in LB broth with Carbenicilin. Plasmids were purified using “E.Z.N.A. Plasmid DNA Mini Kit I (Omega Bio-Tek, D6842-02) and sequence verified through long-read sequencing (Plasmidsaurus). Plasmids used for transfection were purified using NucleoBond Xtra Midi EF (Macherey-Nagel, 740420.50).

### Secretion assay

For INS-1 832/13 cells, 200,000 cells were plated out into individual wells of a 24-well plate (GenClone, 25-107) coated with PLL (Sigma-Aldrich, P2636-500MG). For PC12 cells, 250,000 cells were plated out into individual wells of a 24-well plate coated with PLL. The following day, cells were transfected with 500ng of ANF-emdGFP, NPY-GFP, NPY-mCherry, NPY-sfCherry2, or 250ng of NPY-sfCherry3c along with 1.5µl Lipofectamine 2000 (ThermoFisher, 11668019), 100µl Opti-Mem (ThermoFisher, 22600050), and 400µl Reduced Serum Media (90% RPMI, 10% Complete INS-1 832/13 media or 90% DMEM, 10% Complete PC12 media) according to manufacturer protocol. After 4 hours, transfection mix was replaced with complete INS-1 832/13 or PC12 media to allow for cell recovery. INS-1 832/13 cells were then moved into 5mM glucose INS-1 832/13 media for 18 hours, followed by 2 hours in 1.5mM glucose INS-1 832/13 media. For secretion, INS-1 832/13 cells were incubated with 250µl of either basal KRB (1.5mM glucose, 5.4mM KCl), stimulatory KRB (16.7mM glucose, 40mM KCl), High Glucose KRB (16.7mM glucose, 5.4mM KCl) or High Potassium KRB (1.5mM glucose, 40mM KCl) for the designated duration of secretion. KRB buffers were supplemented with 0.2% fatty-acid free BSA. Forskolin was prepared at 20mM in 100% ethanol and then added to KRB at a final concentration of 10µM. PC12 cells were incubated with 250µl of either basal Tyrode’s (2.5mM KCl) or stimulatory Tyrode’s (90mM KCl). At the end of secretion, the 24-well plate was placed immediately on ice and KRB or Tyrode’s containing secreted reporter was transferred to a 1.5ml tube on ice. Secreted fractions were spun down for 5 minutes at 1000 x g and the supernatant was then transferred to a fresh tube on ice. 250µl lysis buffer (50mM Tris pH8, 150mM NaCl, 1mM EDTA, 1% Triton X-100, 1mM PMSF, 1x Protease Inhibitor) was added to each well, followed by a brief scraping of the well and a 5-minute incubation. Cellular lysates were collected and spun down for 8 minutes at 17,000 x g before being transferred to a new tube. 100µl of each secreted or cellular sample was added to a black 96-well polystyrene plate (Greiner, 655096). Secreted and cellular NPY was determined using Tecan Infinite M1000 plate reader and Tecan i-control software. For all Cherry constructs, excitation was set at 587 nm and emission was set at 610nm with a bandwidth of 10nm for both. The plate was read 4 times per well from the bottom with a gain set at 190. For GFP, excitation was set at 488 nm with 5nm bandwidth and emission was set at 530 nm with 15nm bandwidth. Plate was read 4 times from the bottom with a gain set at 170. Background signal was determined by fluorescent reading of secreted fractions or cellular lysates prepared from untransfected cells and subsequently subtracted from each respective sample.

### Flow cytometry

INS-1 832/13 or PC12 cells were plated in a 24-well plate and transfected with NPY-mCherry, NPY-sfCherry2, or NPY-sfCherry3c as previously described. For INS-1 832/13 cells, experiments were performed 24 hours post-transfection, and for PC12 cells, 48 hours post-transfection. Cells were transferred from their wells to a 1.5ml centrifuge tube (Olympus Plastics, 24-282) and centrifuged at 0.3 x g for 5 minutes. The media was carefully removed without disturbing the pellet, which was then resuspended in 100µl 4% paraformaldehyde (PFA) (Sigma-Aldrich, P6148-500g) in 1x PBS (VWR, 76361-734). After 20 minutes of fixation, 1ml of 1x PBS was added to each tube and mixed thoroughly. Cells were pelleted again by centrifugation at 1000 x g for 5 minutes. The supernatant was carefully removed, and the cell pellet was resuspended in 250µl 1x PBS. Flow cytometry analysis was conducted using a BD Accuri C6 Plus Flow Cytometer, with gates set based on untransfected WT cells.

### Western blot

INS-1 832/13 cells were plated in a 24-well plate and transfected with NPY-mCherry, NPY-sfCherry2, or NPY-sfCherry3c as described above. After 48 hours, cells were placed on ice and lysed in 250µl lysis buffer for 5 minutes. Cellular lysates were collected and spun down for 8 minutes at 17,000 x g in microfuge and then transferred to a new tube. 45µl of cellular lysate was combined with 15µl of 4x Sample Buffer and 3µl of 1M DTT (GoldBio, DTT10). SDS-PAGE was performed using a 10% gel. Following a 1 hour transfer onto a 0.45µm nitrocellulose membrane (Bio-Rad, 1620115), blocking was done using 5% milk in PBS-T (VWR, M147-1L). The membrane was then immunblotted for 1 hour at room temperature using rabbit NPY (Cell Signaling, 11976) at 1:1000 in PBS-T milk or mouse Actin (DSHB, JLA20) at 1:100 in PBS-T milk. The membrane was then washed 3 times in PBS-T for 5 minutes each wash followed by immunoblotting with anti-mouse-AlexaFluor790 antibody (ThermoFisher, A-11357) at 1:5000 in PBS-T milk or anti-rabbit-AlexaFluor647 (ThermoFisher, A-21245) at 1:1000 in PBS-T milk for 1 hour at room temperature. The membrane was washed 3 more times in PBS-T prior to imaging using ProteinSimple FluorChem R imager. Images were analyzed and quantified using ImageJ.

### Equilibrium gradient

INS-1 832/13 cells were cultured in a 15cm dish (GenClone, 25-203) and transfected using 15ug NPY-sfCherry3c, 83µl PEI (Sigma-Aldrich, 408700), 5mL Opti-mem, and 20mL of Reduced Serum Media for 48 hours. Continuous sucrose gradients were prepared in Ultra-Clear centrifuge tubes (Beckman Coulter, 344059) coated using Sigmacote (Sigma-Aldrich) using 0.6M Sucrose, 10mM HEPES and 1.6M Sucrose, 10mM HEPES. NPY-sfCherry3c transfected cells were harvested from the 15cm plate using 10mL of PBS and spun down for 5 minutes at 0.3 x g. PBS was aspirated and the cell pellet was resuspended in 5mL of SH buffer (0.3M Sucrose, 10mM HEPES) and spun down for 5 minutes at 0.3 x g. SH buffer was aspirated and the cell pellet was resuspended in 750µl SH buffer. Cells were carefully needled using 1 mL syringe (BD, 309628) with a 22G needle (BD, 305156) to break up cell clumps. Cells were then pushed through a cell homogenizer (Isobiotec) with an 18µm clearance ten times to break open cells. Cracked cell lysate was then collected and spun down for 8 minutes at 1000 x g. Post-Nuclear Supernatant (PNS) was collected and carefully added to the top of the gradient. The gradient was spun down in an Optima LE-80k Ultracentrifuge with a SW41 rotor (Beckman Coulter) for 16 hours at 30,000 x g in a vacuum at 4C. The following day, 500µl fractions were collected from the top of the gradient and 100µl of each fraction was loaded onto a 96-well polystyrene plate (Greiner, 655096) along with triplicates of SH buffer, 0.6M Sucrose, and 1.6M Sucrose for background controls. The plate was read using Tecan Infinite M1000 plate reader and Tecan i-control software using the same settings as described above.

### Microscopy

INS-1 832/13 or PC12 cells were plated out and transfected in a 24-well plate as previously described. The following day, cells were transferred to glass coverslips (Warner Instruments, 64-0718). After an additional 24 hours, cells were fixed in 4% PFA in 1x PBS for 20 minutes, washed 3 times with PBS, and quenched for 10 minutes in 100mM ammonium chloride (Sigma-Aldrich, A9434-500G). Cells were then incubated in 250µl Block Buffer composed of 1% fish scale gelatin (Sigma-Aldrich, G7041-500G), 2% fatty-acid-free BSA, (GoldBio, A-421-100), and 0.02% Saponin (Sigma-Aldrich, S7900) in 1x PBS for one hour. INS-1 832/13 cells were then incubated with a mouse insulin antibody (Sigma, I2018) at 1:1000 dilution in block buffer, while PC12 cells were incubated with a rabbit SgII antibody (Meridian Bioscience, K55101R) at 1:1000 in block buffer for one hour. After antibody incubation, cells were rinsed three times in block buffer and incubated with an anti-mouse-AlexaFluor647 antibody (ThermoFisher, A-21235) at 1:1000 for INS-1 832/13 cells or an anti-rabbit-AlexaFluor647 at 1:1000 (ThermoFisher, A-21245) for PC12 cells for one hour. Following another 3 rinses, coverslips were mounted on glass slides (Fisher Scientific, 12-544-1) using ProLong Gold antifade mounting media (Invitrogen, P36934). The coverslips were imaged using an EVOS FL Auto 2 microscope (Invitrogen, AMAFD2000) with a PlanAPO N 60x, 1.42NA objective lens. Images were deconvoluted using the ImageJ plugin DeconvolutionLab2 with the Richardson-Lucy algorithm for 30 iterations. For TIRF microscopy, cells were imaged using a custom built TIRF microscope (Alpha-Plan APO 60x, 1.49 NA). For live TIRF microscopy, cells were imaged every 0.2 seconds. Cells were imaged for 15 seconds in basal KRB and then stimulated and imaged for 60 seconds in high potassium KRB.

### Statistics

All statistical analysis was performed using one-way or two-way ANOVA followed by Tukey’s multiple comparisons test. Statistical analyses were conducted using Excel or Prism.

## Data Availability

The original contributions presented in the study are included in the article/**Supplementary Material**. Further inquiries can be directed to the corresponding author.

## Supplementary material

Supplementary Figure 1
**(A)** Histograms of untransfected INS-1 832/13 cells or NPY-mCherry, NPY-sfCherry2, and NPY-sfCherry3c transfected INS-1 832/13 cells.

Supplementary Figure 2
**(A, B)** Bar graphs from **Figures 2A** or **2B** with indicated significance values by two-way ANOVA. *p<0.05, **p<0.01, ***p<0.001, ****p<0.0001.

Supplementary Figure 3
**(A)** Histograms of untransfected PC12 or NPY-mCherry, NPY-sfCherry2, and NPY-sfCherry3c transfected PC12 cells.

Movie S1 Live movie of an NPY-sfCherry3c transfected INS-1 832/13 cell in TIRF undergoing exocytosis. Images were taken every 200ms. 5 seconds of cell in basal KRB followed by 25 seconds of cell in high K+ KRB.

Supplementary Table 1 Sequence of gene blocks used to clone NPY and the respective indicated fluorescent reporters.

Supplementary Table 2 *Detailed cost breakdown for ELISA vs NPY secretion assay. As one ELISA kit costs $678.50 and has a max loading capacity of 36 samples (loaded in duplicates), each sample costs $18.85. In contrast, using the fluorescence plate reader-based secretion assay, the cost per sample comes down to $1.50 ($0.72 for the plate and $0.78 for the transfection), which represents a ~12.5x cost reduction per sample compared to ELISA. The 316 samples used for this study would have required a total of 9 ELISA plates, bringing the total cost to $6106.50. In contrast, the total cost actually incurred for this study by using this newly developed secretion assay is only ~$478.
