# Supplementary figures and images for: An optimized fluorescent reporter enables rapid and cost-effective quantification of regulated secretion from neuroendocrine cells
Source: Front Endocrinol (Lausanne). 2025 Aug 18;16:1640601. doi: 10.3389/fendo.2025.1640601 (PMC12400516; doi:10.3389/fendo.2025.1640601)

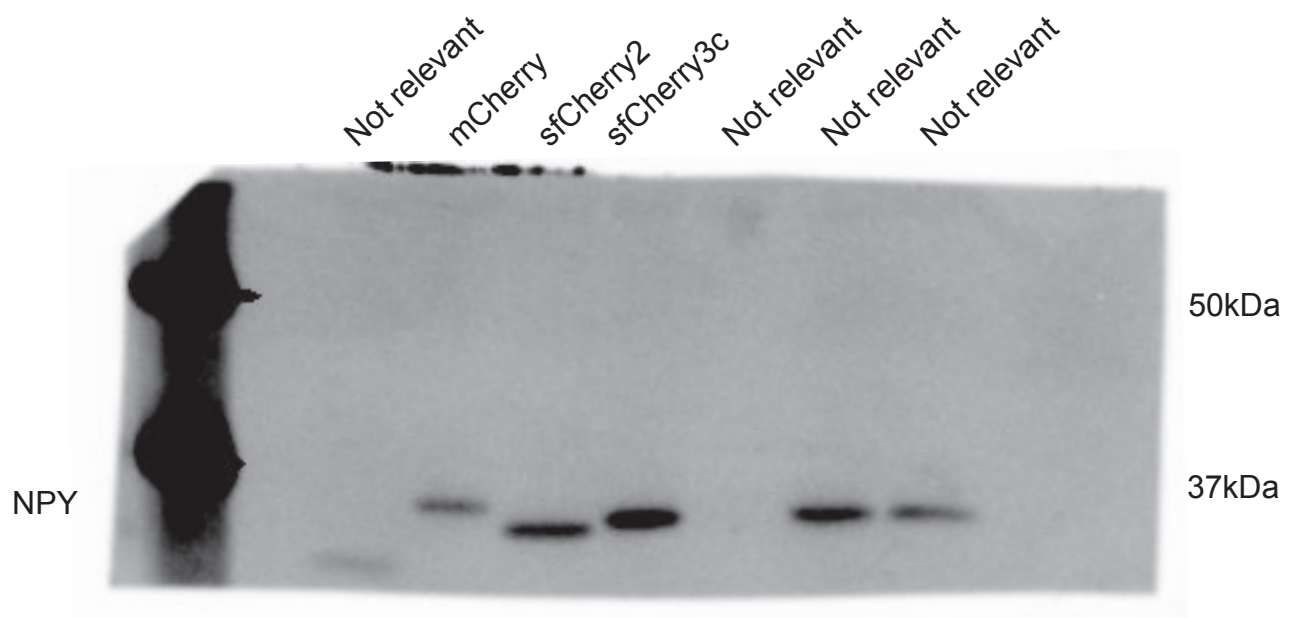

Nonspecific

Actin

42kDa

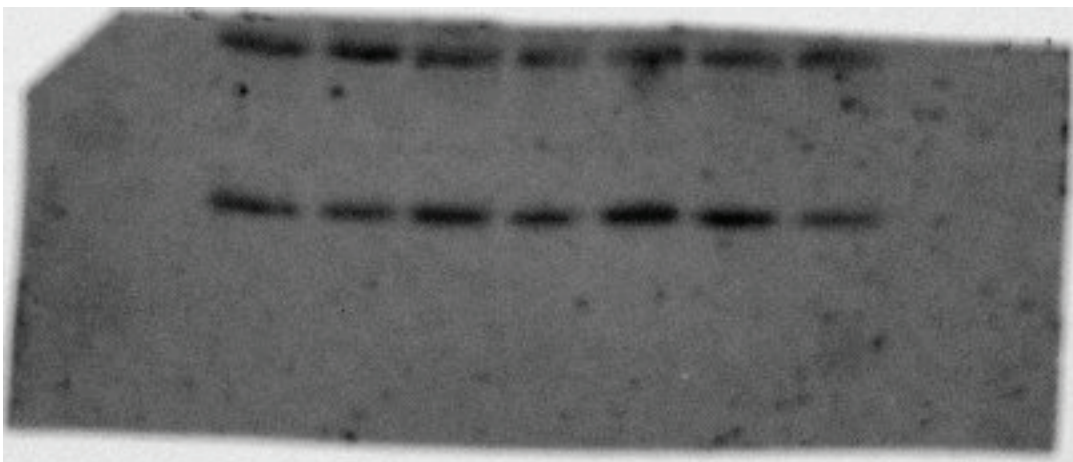

Supplement: Supplementary Figure 1 — (A) Histograms of untransfected INS-1 832/13 cells or NPY-mCherry, NPY-sfCherry2, and NPY-sfCherry3c transfected INS-1 832/13 cells. [file DataSheet1.pdf]

A)

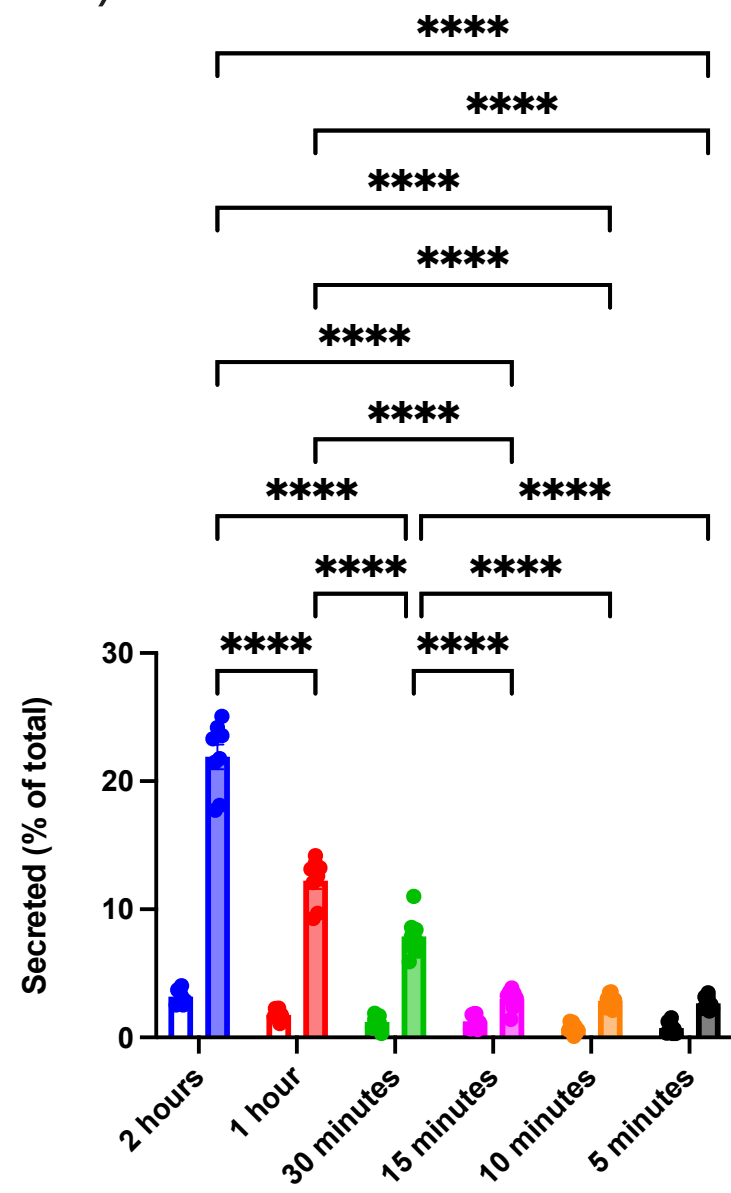

B)

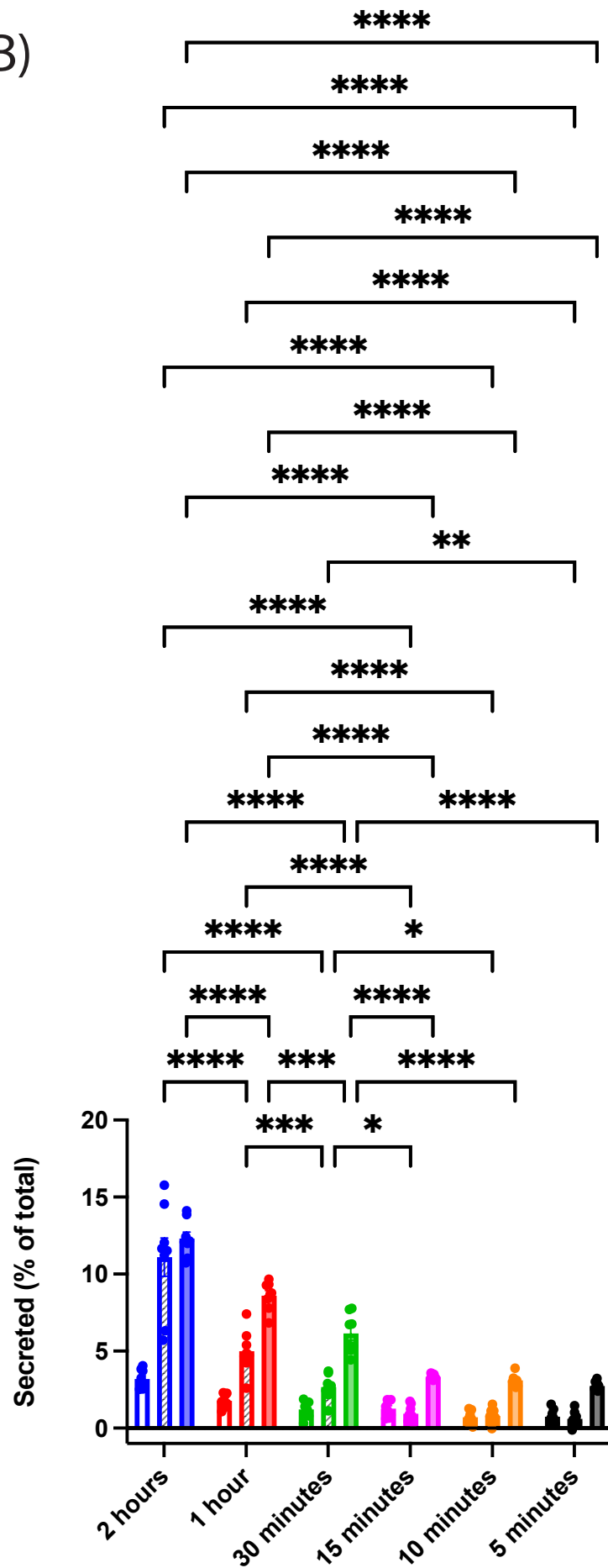

Supplement: Supplementary Figure 2 — (A, B) Bar graphs from Figures 2A or 2B with indicated significance values by two-way ANOVA. *p<0.05, **p<0.01, ***p<0.001, ****p<0.0001. [file DataSheet2.pdf]

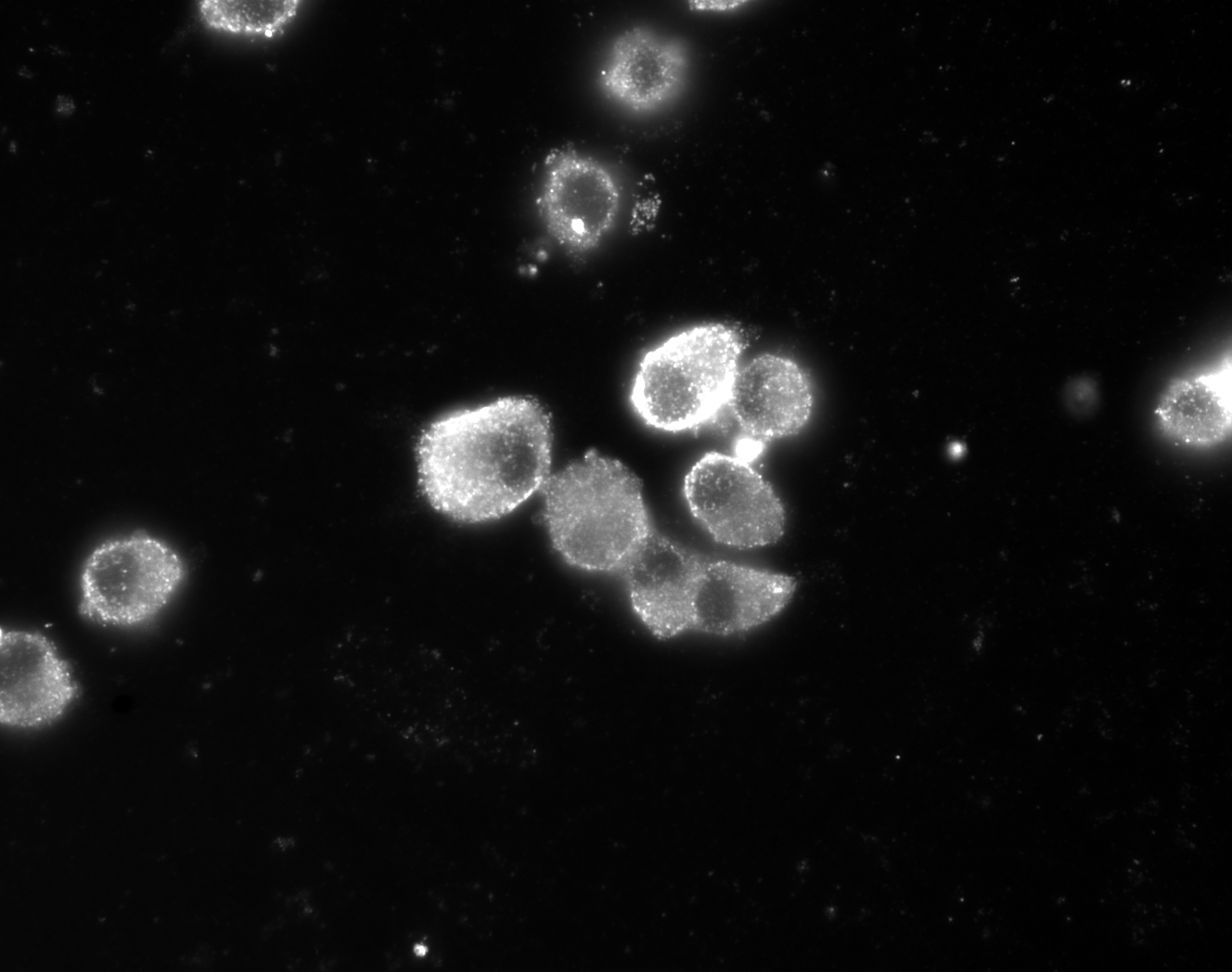

Supplement: Supplementary Figure 3 — (A) Histograms of untransfected PC12 or NPY-mCherry, NPY-sfCherry2, and NPY-sfCherry3c transfected PC12 cells. [file DataSheet3.zip › 4b/mCherry/SGII.tif]

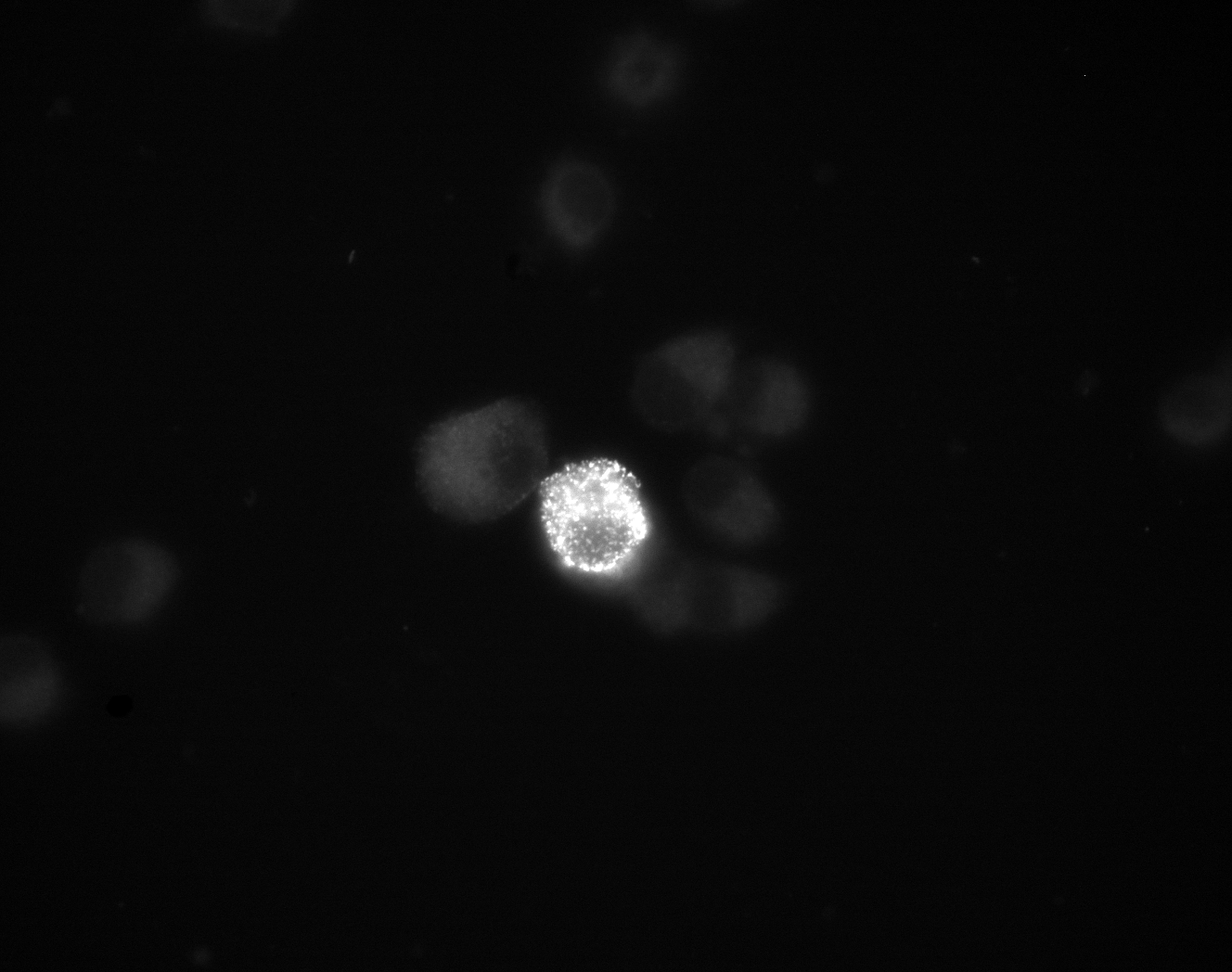

Supplement: Supplementary Figure 3 — (A) Histograms of untransfected PC12 or NPY-mCherry, NPY-sfCherry2, and NPY-sfCherry3c transfected PC12 cells. [file DataSheet3.zip › 4b/mCherry/NPY.tif]

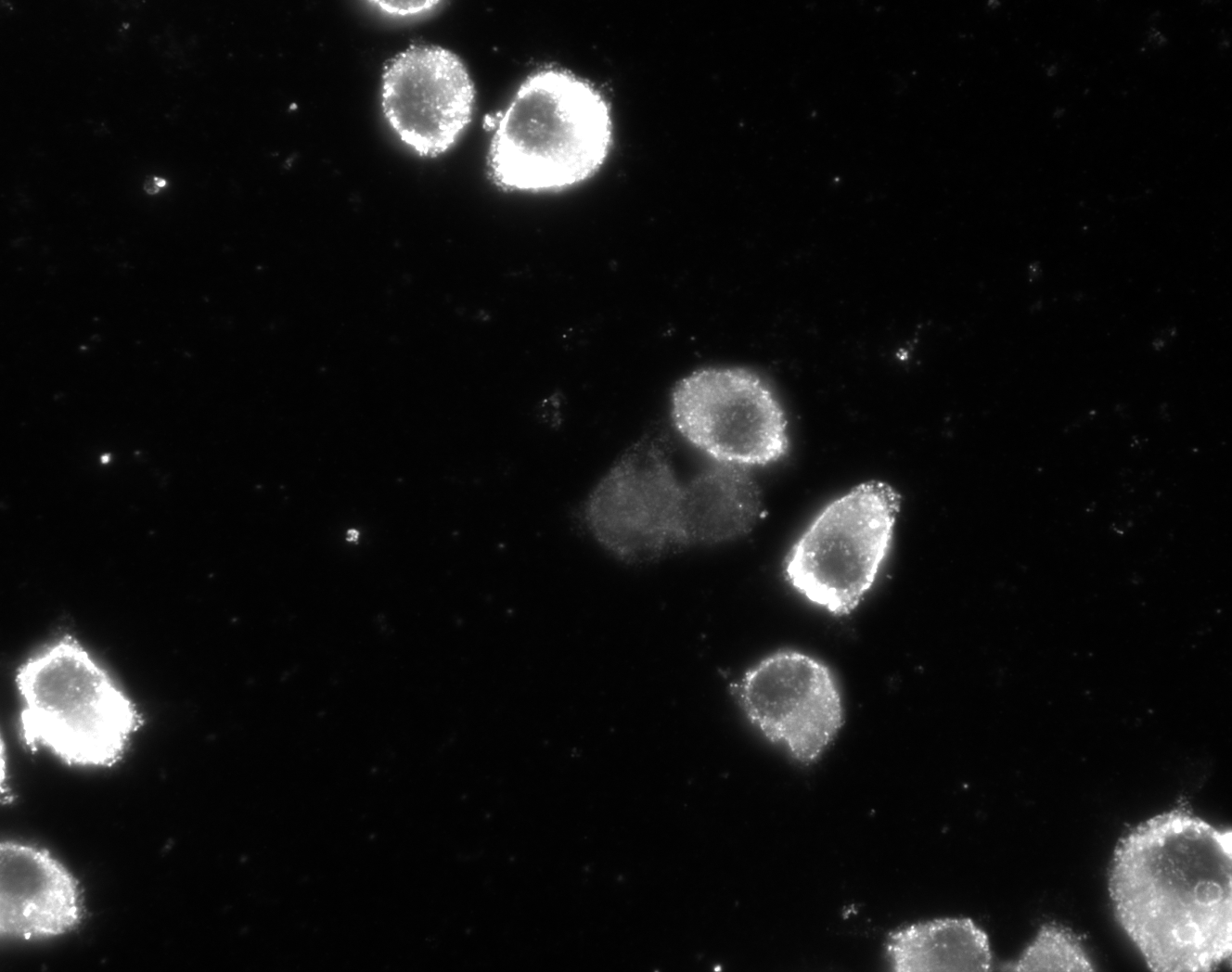

Supplement: Supplementary Figure 3 — (A) Histograms of untransfected PC12 or NPY-mCherry, NPY-sfCherry2, and NPY-sfCherry3c transfected PC12 cells. [file DataSheet3.zip › 4b/sfCherry2/SGII.tif]

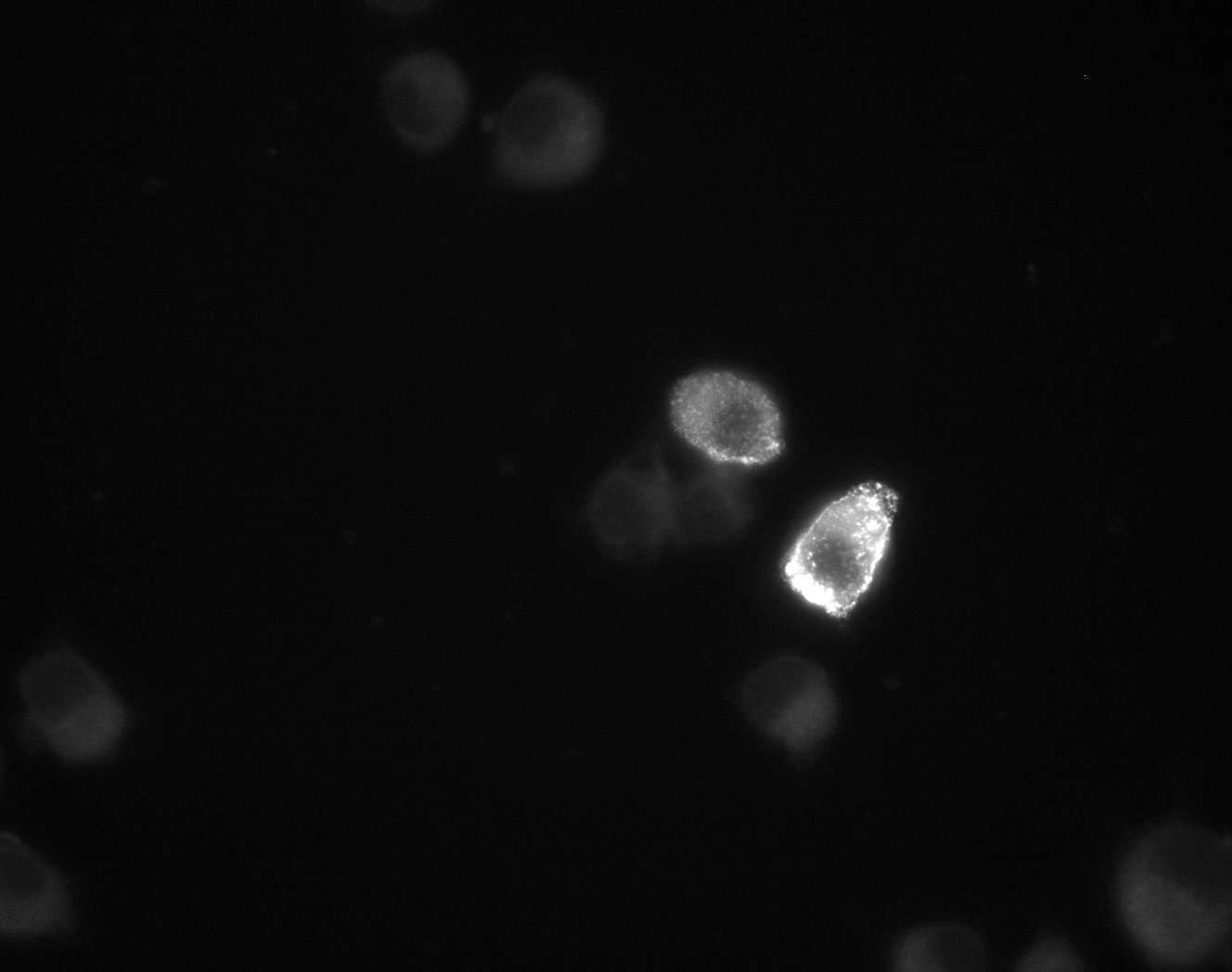

Supplement: Supplementary Figure 3 — (A) Histograms of untransfected PC12 or NPY-mCherry, NPY-sfCherry2, and NPY-sfCherry3c transfected PC12 cells. [file DataSheet3.zip › 4b/sfCherry2/NPY.tif]

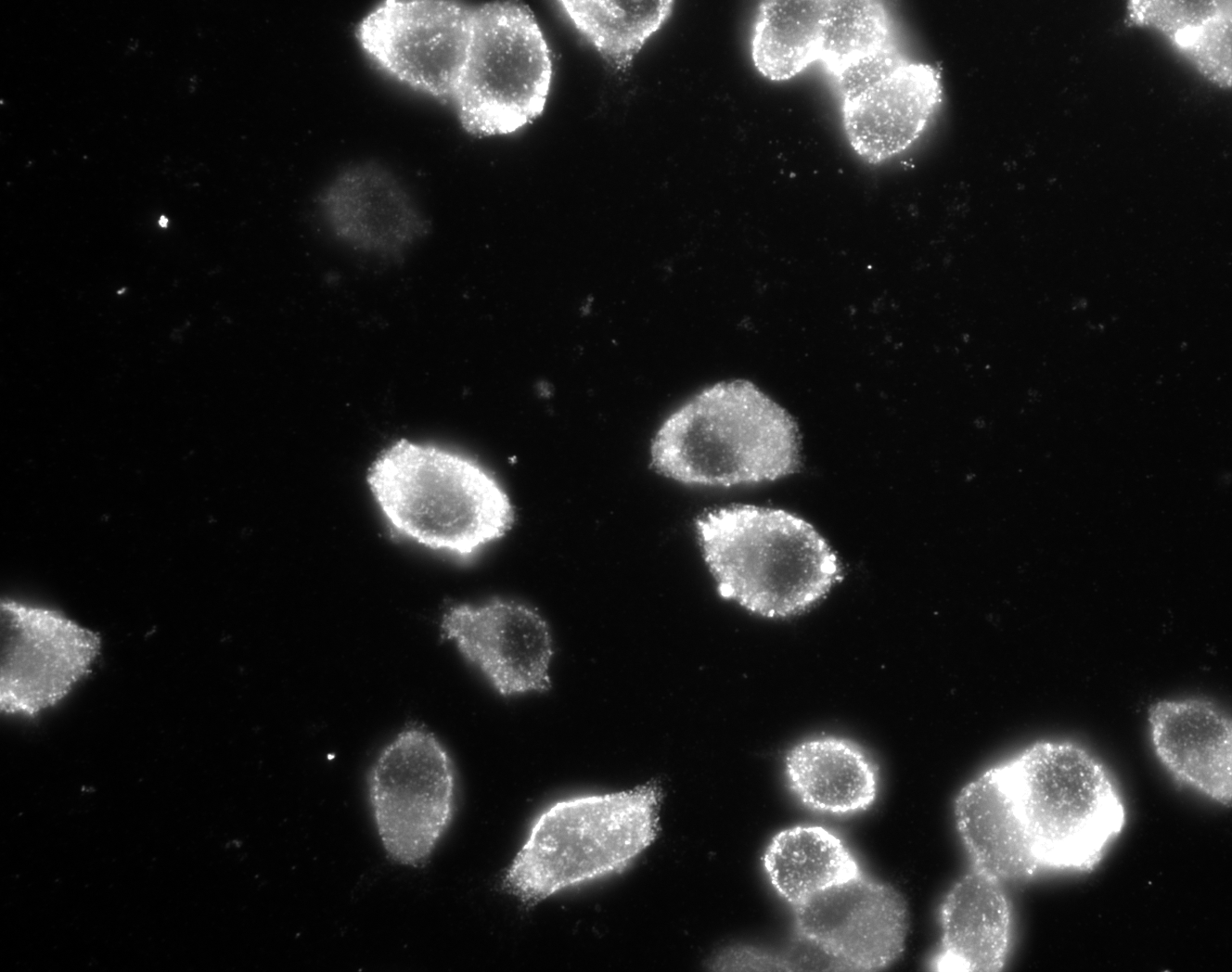

Supplement: Supplementary Figure 3 — (A) Histograms of untransfected PC12 or NPY-mCherry, NPY-sfCherry2, and NPY-sfCherry3c transfected PC12 cells. [file DataSheet3.zip › 4b/sfCherry3c/SGII.tif]

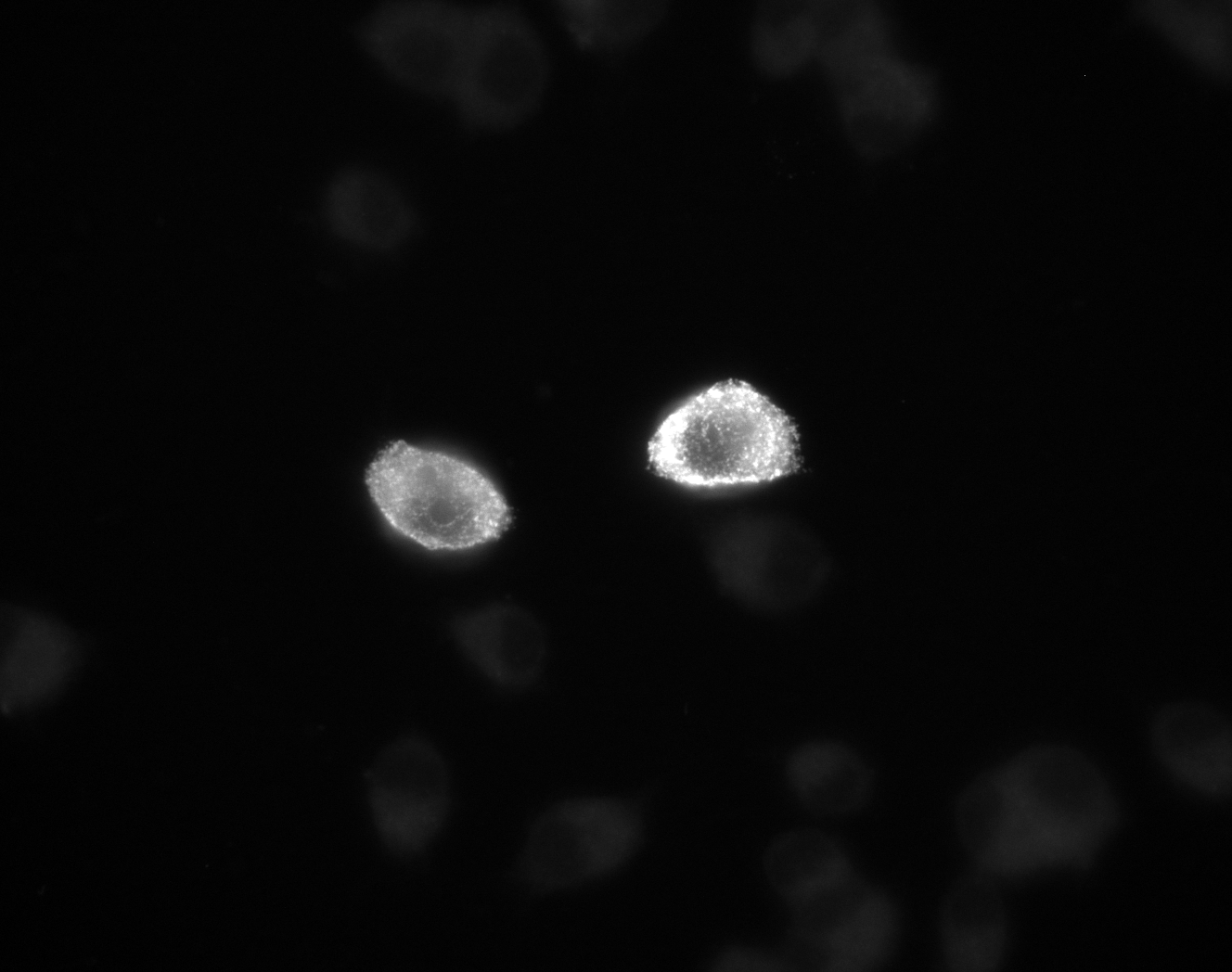

Supplement: Supplementary Figure 3 — (A) Histograms of untransfected PC12 or NPY-mCherry, NPY-sfCherry2, and NPY-sfCherry3c transfected PC12 cells. [file DataSheet3.zip › 4b/sfCherry3c/NPY.tif]

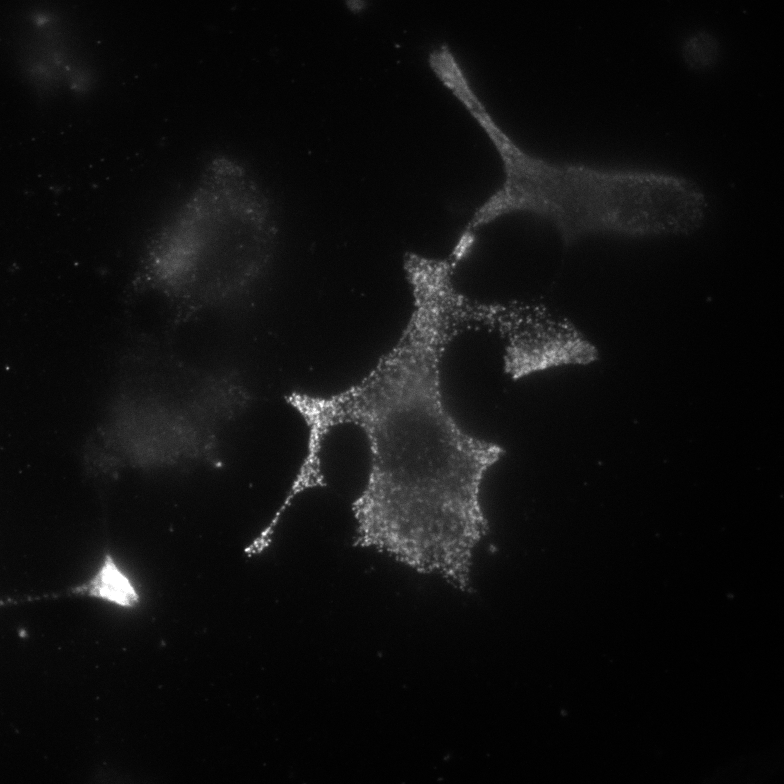

Supplement: Supplementary Figure 3 — (A) Histograms of untransfected PC12 or NPY-mCherry, NPY-sfCherry2, and NPY-sfCherry3c transfected PC12 cells. [file DataSheet3.zip › 1d/mCherry/mCherry.tif]

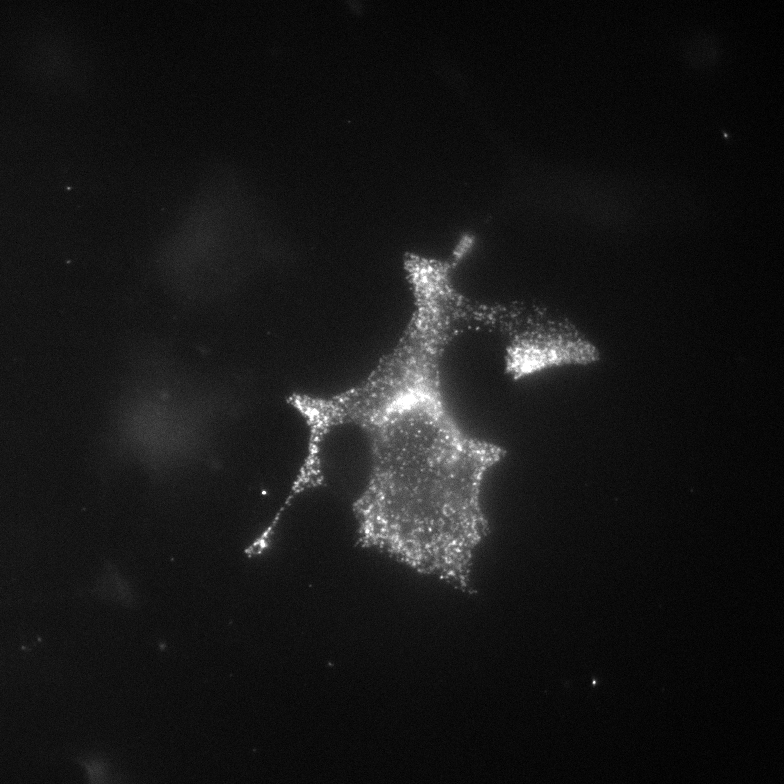

Supplement: Supplementary Figure 3 — (A) Histograms of untransfected PC12 or NPY-mCherry, NPY-sfCherry2, and NPY-sfCherry3c transfected PC12 cells. [file DataSheet3.zip › 1d/mCherry/Insulin.tif]

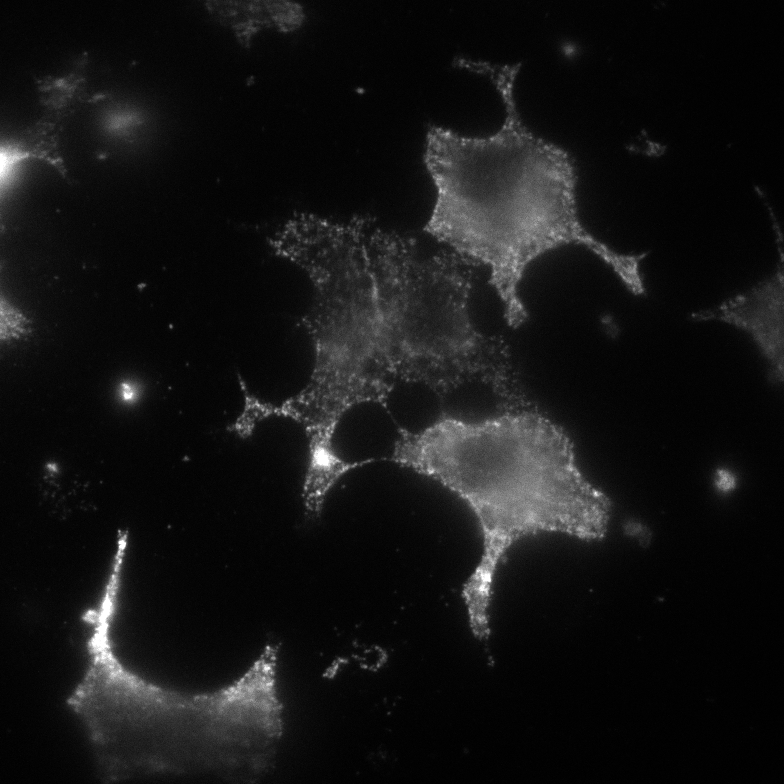

Supplement: Supplementary Figure 3 — (A) Histograms of untransfected PC12 or NPY-mCherry, NPY-sfCherry2, and NPY-sfCherry3c transfected PC12 cells. [file DataSheet3.zip › 1d/sfCherry2/sfCherry2.tif]

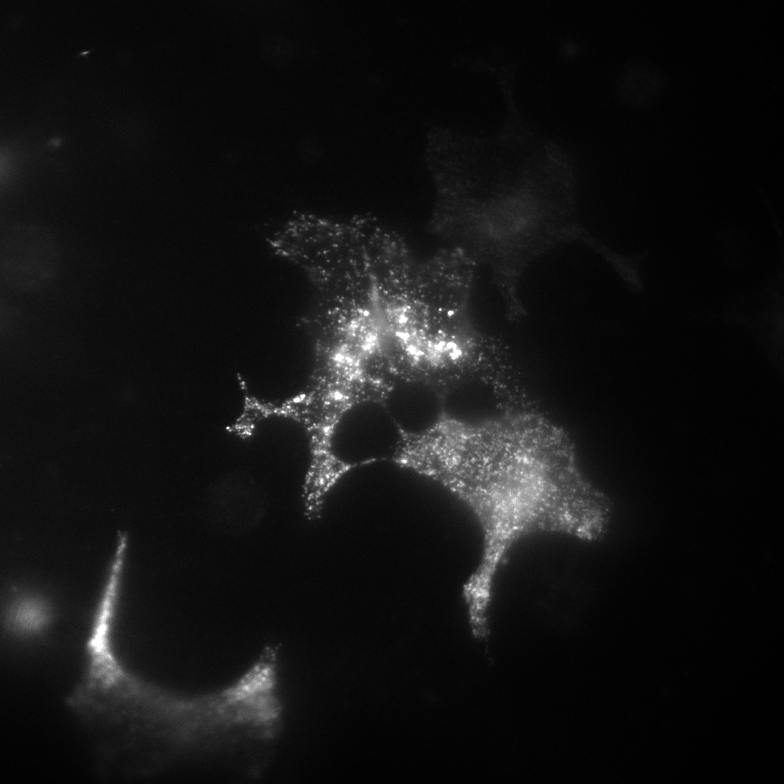

Supplement: Supplementary Figure 3 — (A) Histograms of untransfected PC12 or NPY-mCherry, NPY-sfCherry2, and NPY-sfCherry3c transfected PC12 cells. [file DataSheet3.zip › 1d/sfCherry2/Insulin.tif]

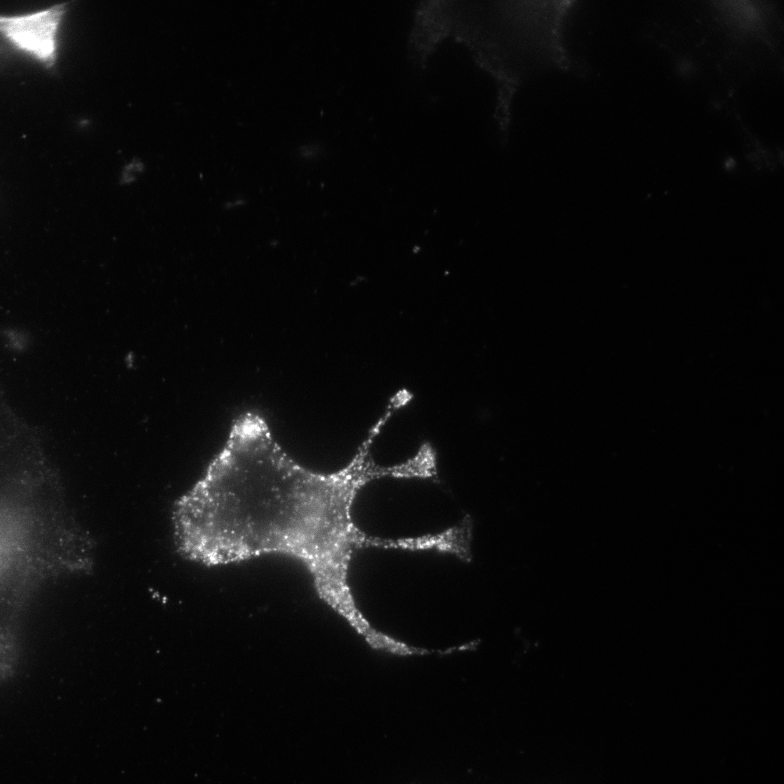

Supplement: Supplementary Figure 3 — (A) Histograms of untransfected PC12 or NPY-mCherry, NPY-sfCherry2, and NPY-sfCherry3c transfected PC12 cells. [file DataSheet3.zip › 1d/sfCherry3c/sfCherry3c.tif]

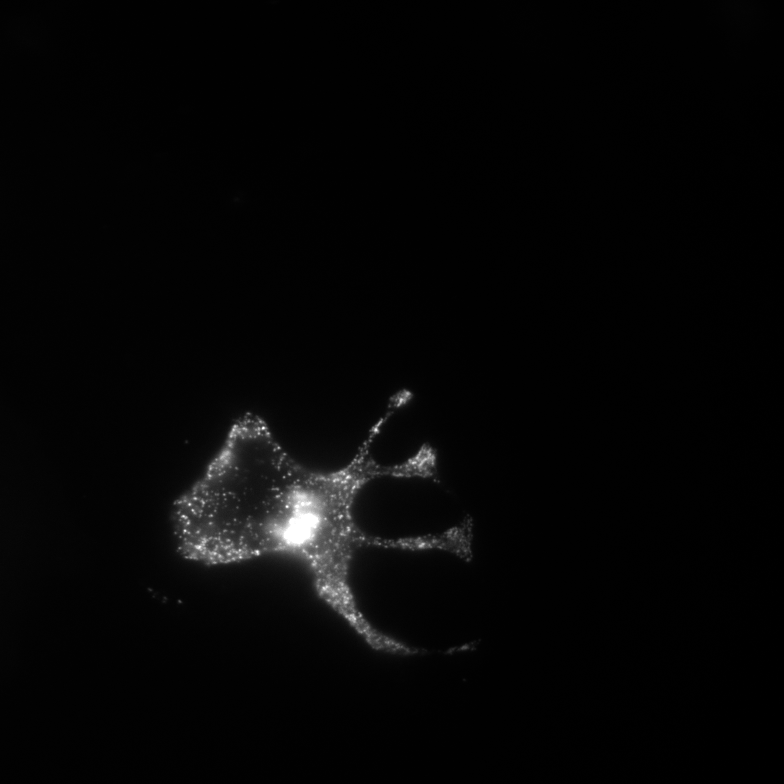

Supplement: Supplementary Figure 3 — (A) Histograms of untransfected PC12 or NPY-mCherry, NPY-sfCherry2, and NPY-sfCherry3c transfected PC12 cells. [file DataSheet3.zip › 1d/sfCherry3c/Insulin.tif]

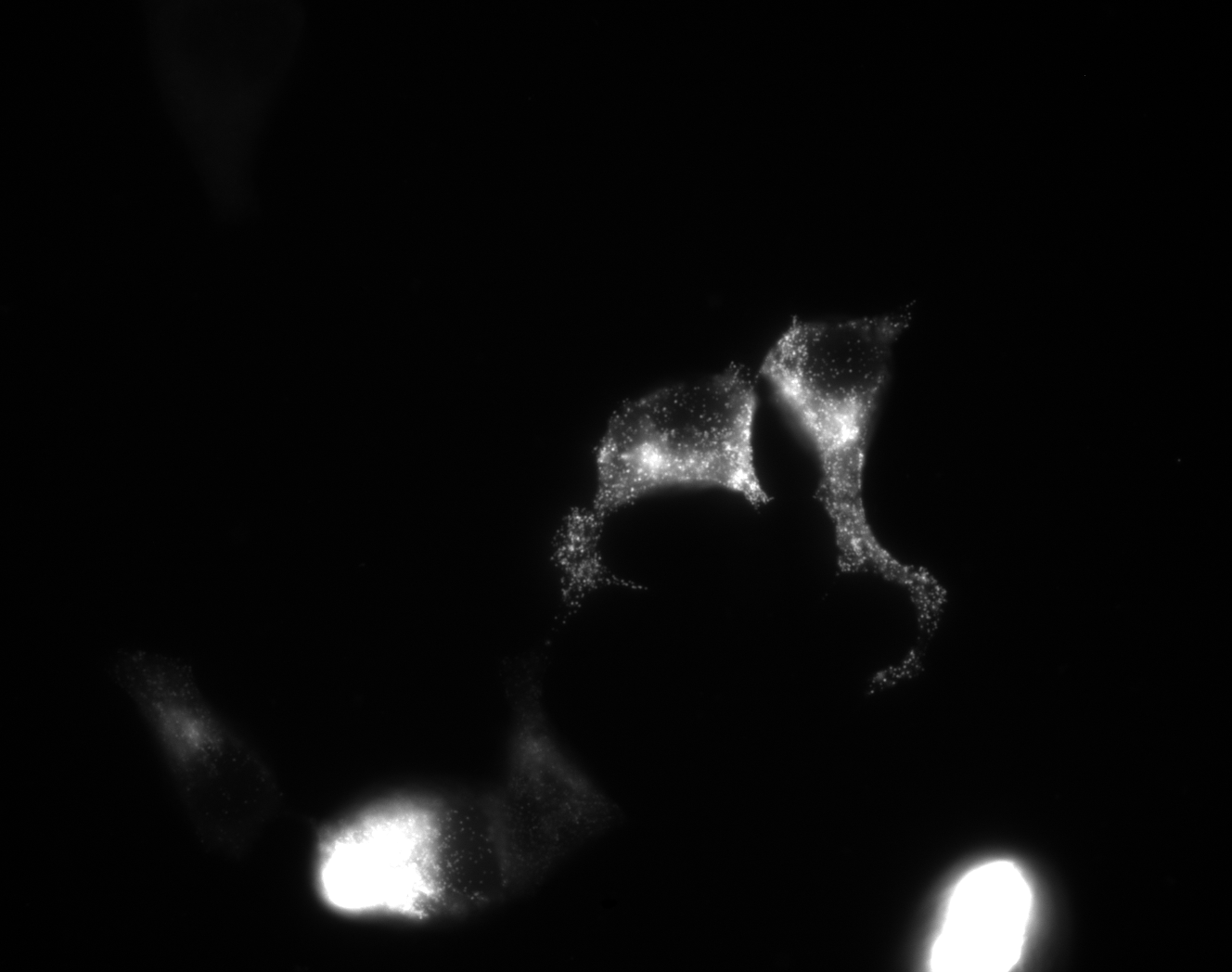

Supplement: Supplementary Figure 3 — (A) Histograms of untransfected PC12 or NPY-mCherry, NPY-sfCherry2, and NPY-sfCherry3c transfected PC12 cells. [file DataSheet3.zip › 1c/mCherry/mCherry.tif]

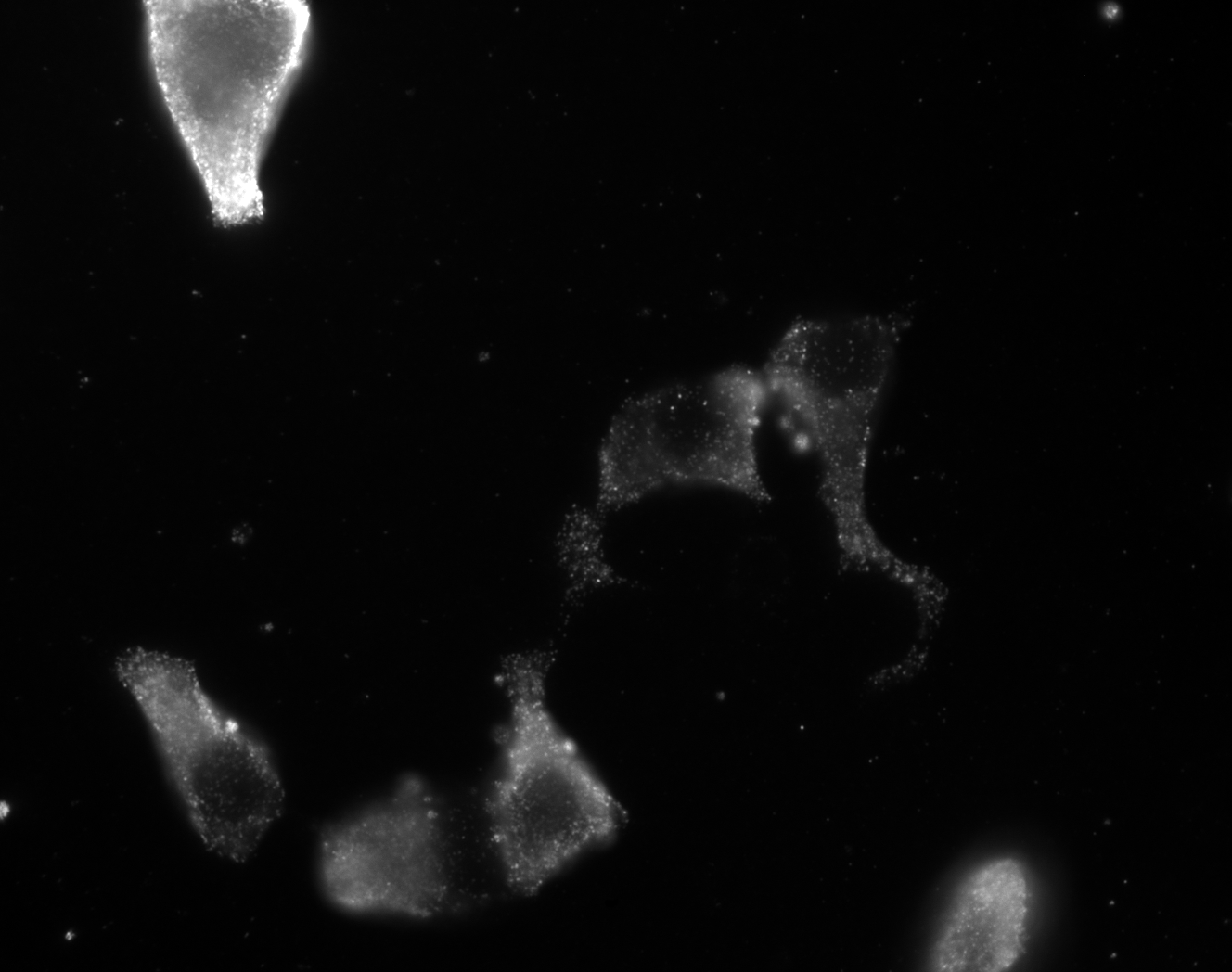

Supplement: Supplementary Figure 3 — (A) Histograms of untransfected PC12 or NPY-mCherry, NPY-sfCherry2, and NPY-sfCherry3c transfected PC12 cells. [file DataSheet3.zip › 1c/mCherry/Insulin.tif]

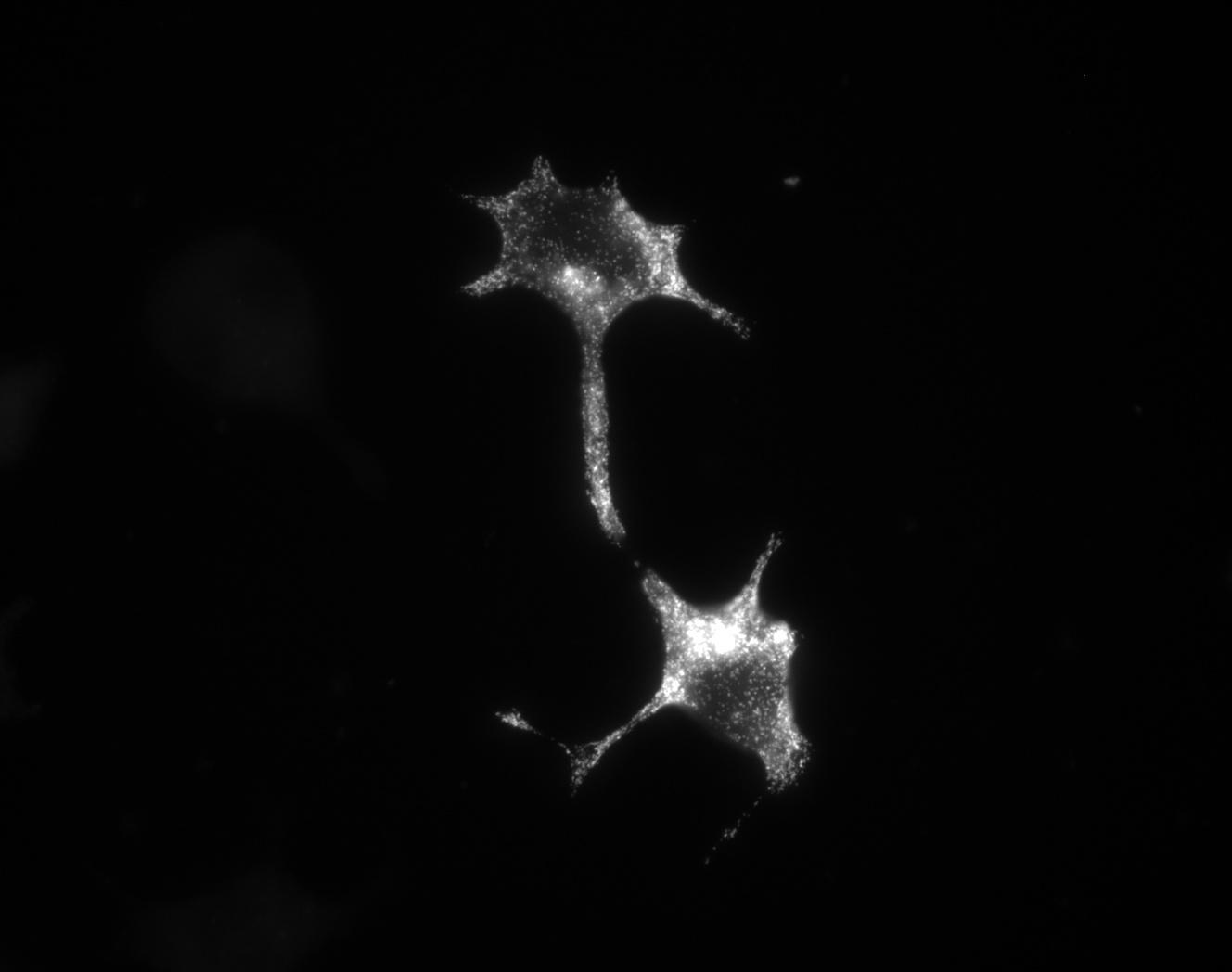

Supplement: Supplementary Figure 3 — (A) Histograms of untransfected PC12 or NPY-mCherry, NPY-sfCherry2, and NPY-sfCherry3c transfected PC12 cells. [file DataSheet3.zip › 1c/sfCherry2/NPY.tif]

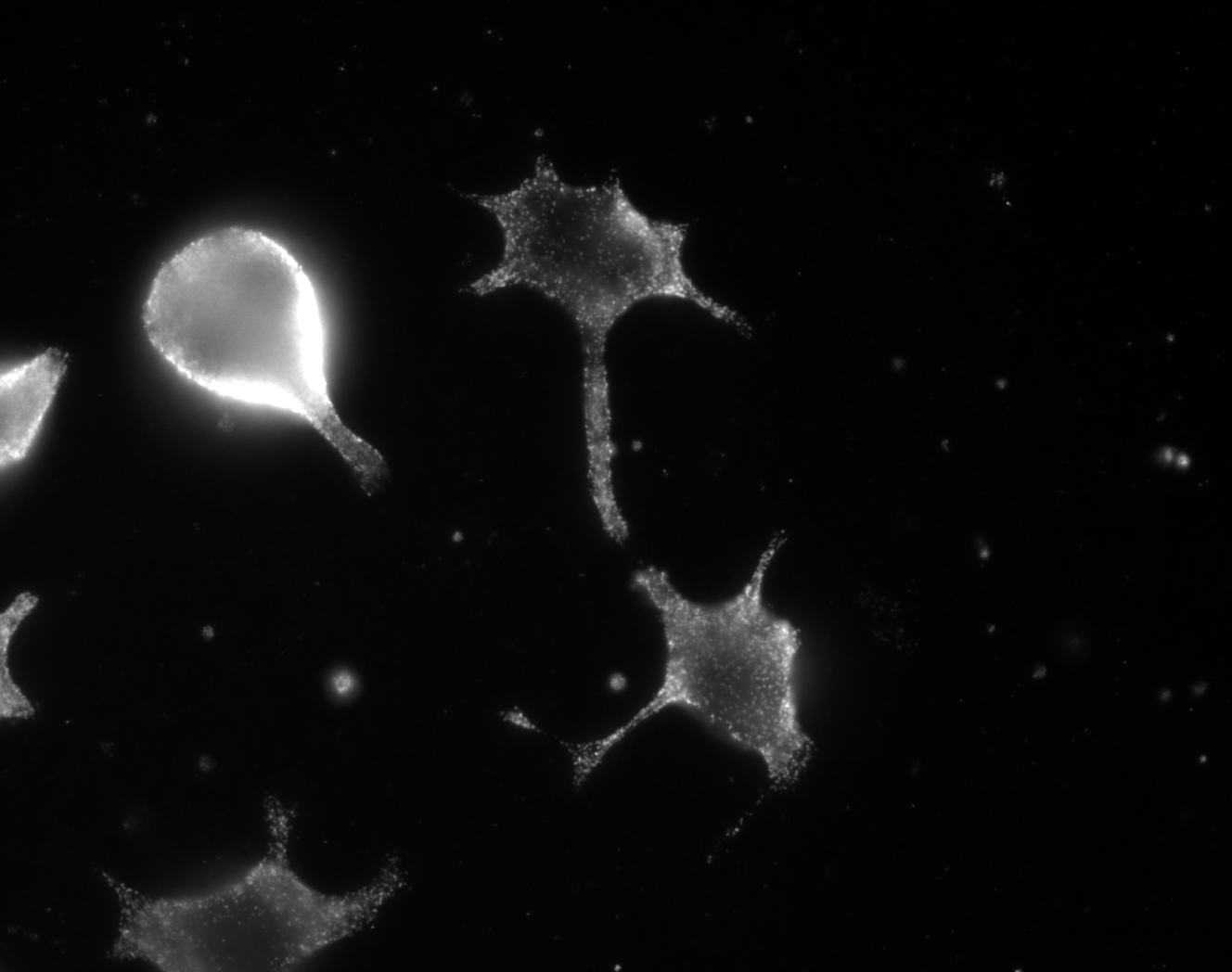

Supplement: Supplementary Figure 3 — (A) Histograms of untransfected PC12 or NPY-mCherry, NPY-sfCherry2, and NPY-sfCherry3c transfected PC12 cells. [file DataSheet3.zip › 1c/sfCherry2/Insulin.tif]

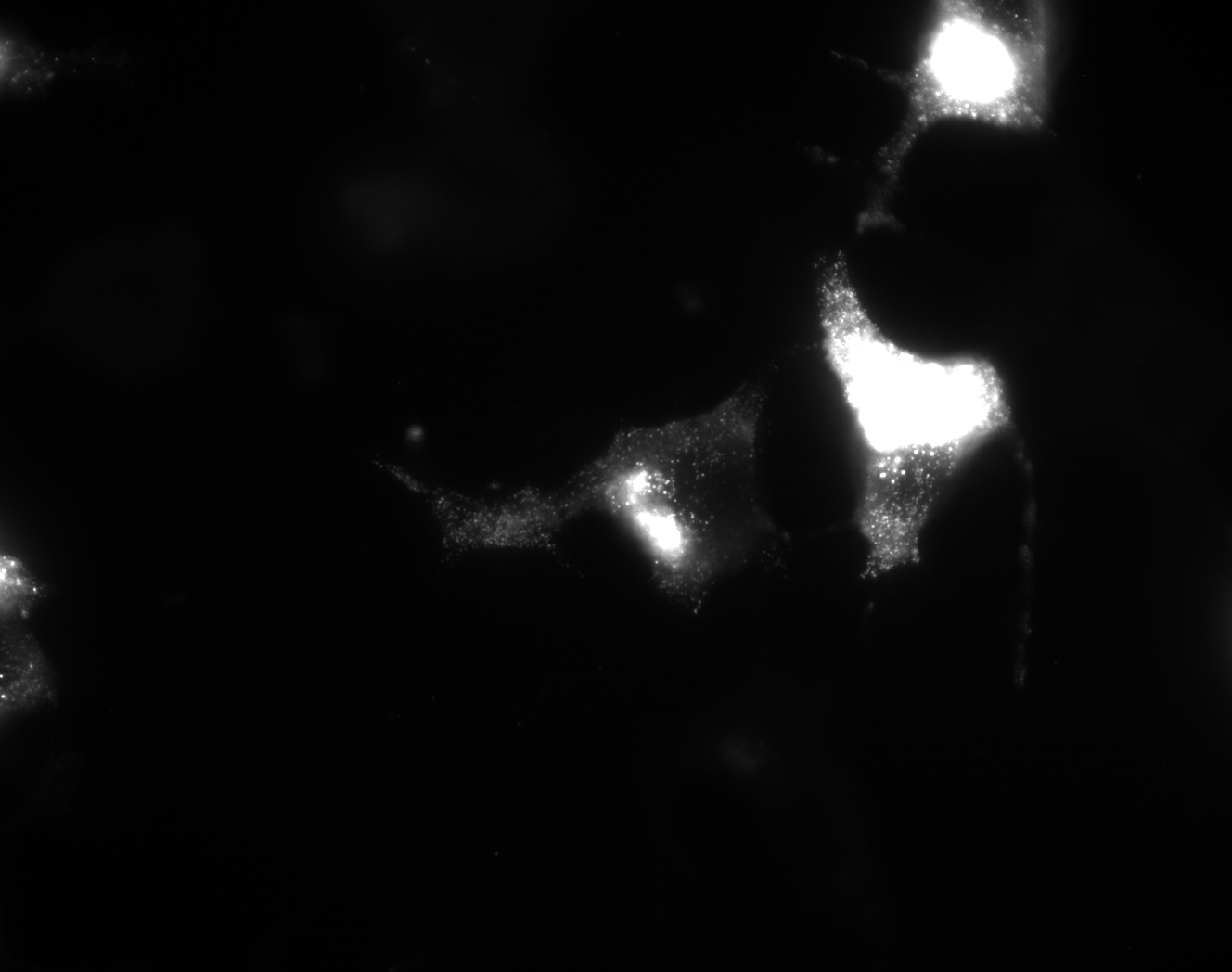

Supplement: Supplementary Figure 3 — (A) Histograms of untransfected PC12 or NPY-mCherry, NPY-sfCherry2, and NPY-sfCherry3c transfected PC12 cells. [file DataSheet3.zip › 1c/sfCherry3c/NPY.tif]

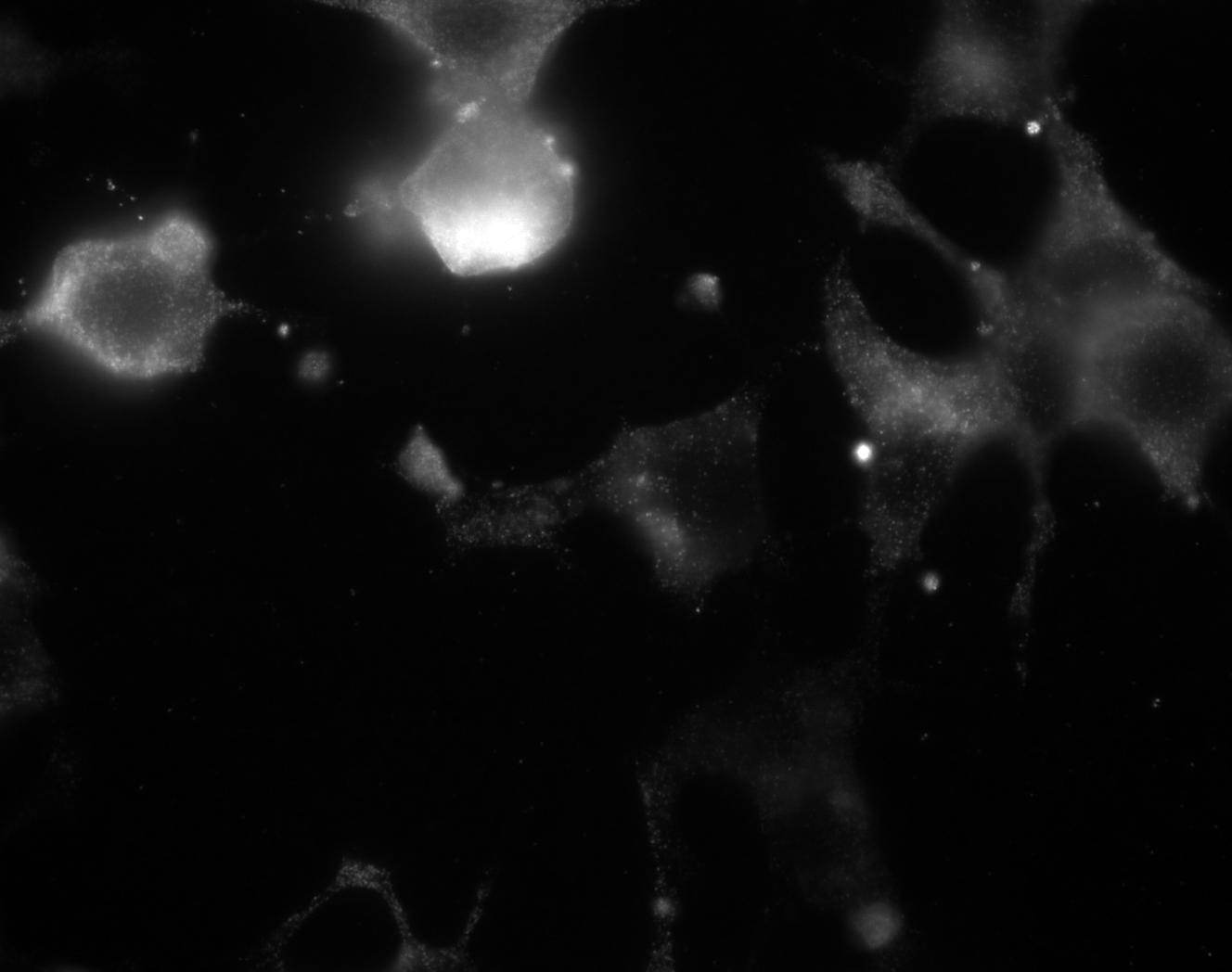

Supplement: Supplementary Figure 3 — (A) Histograms of untransfected PC12 or NPY-mCherry, NPY-sfCherry2, and NPY-sfCherry3c transfected PC12 cells. [file DataSheet3.zip › 1c/sfCherry3c/Insulin.tif]

A)

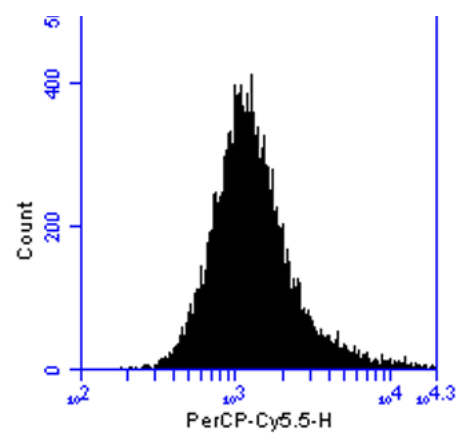

Untransfected

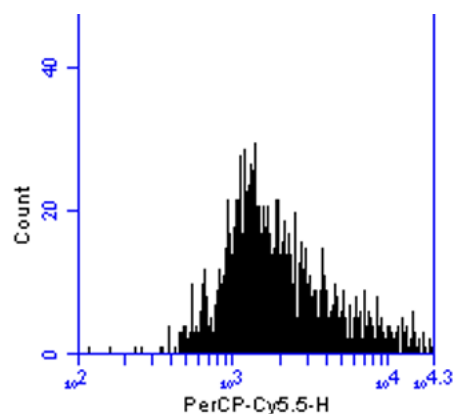

NPY-mCherry

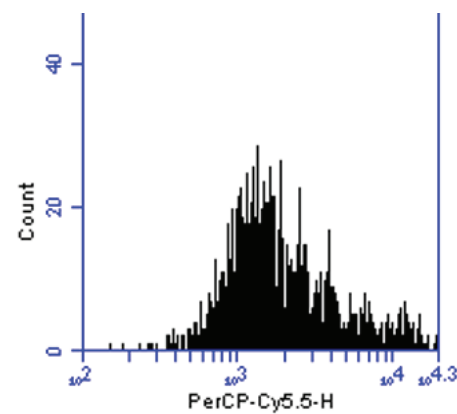

NPY-sfCherry2

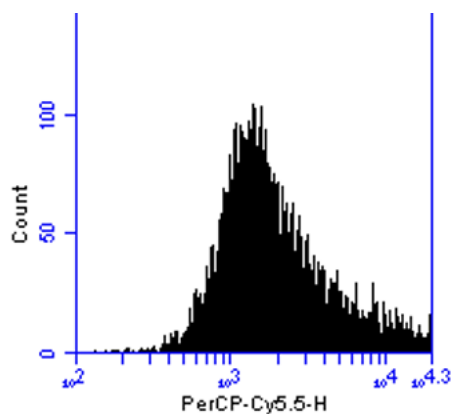

NPY-sfCherry3c

Supplement: Supplementary file 5 [file DataSheet5.pdf]

A)

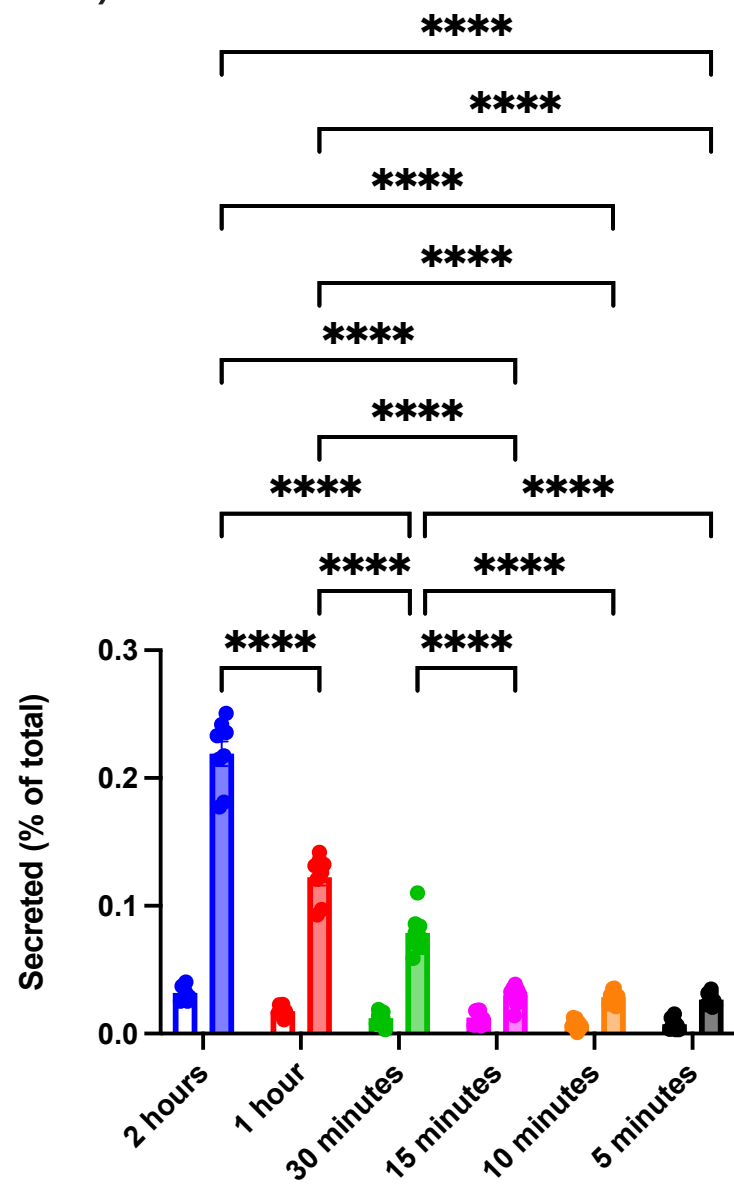

B)

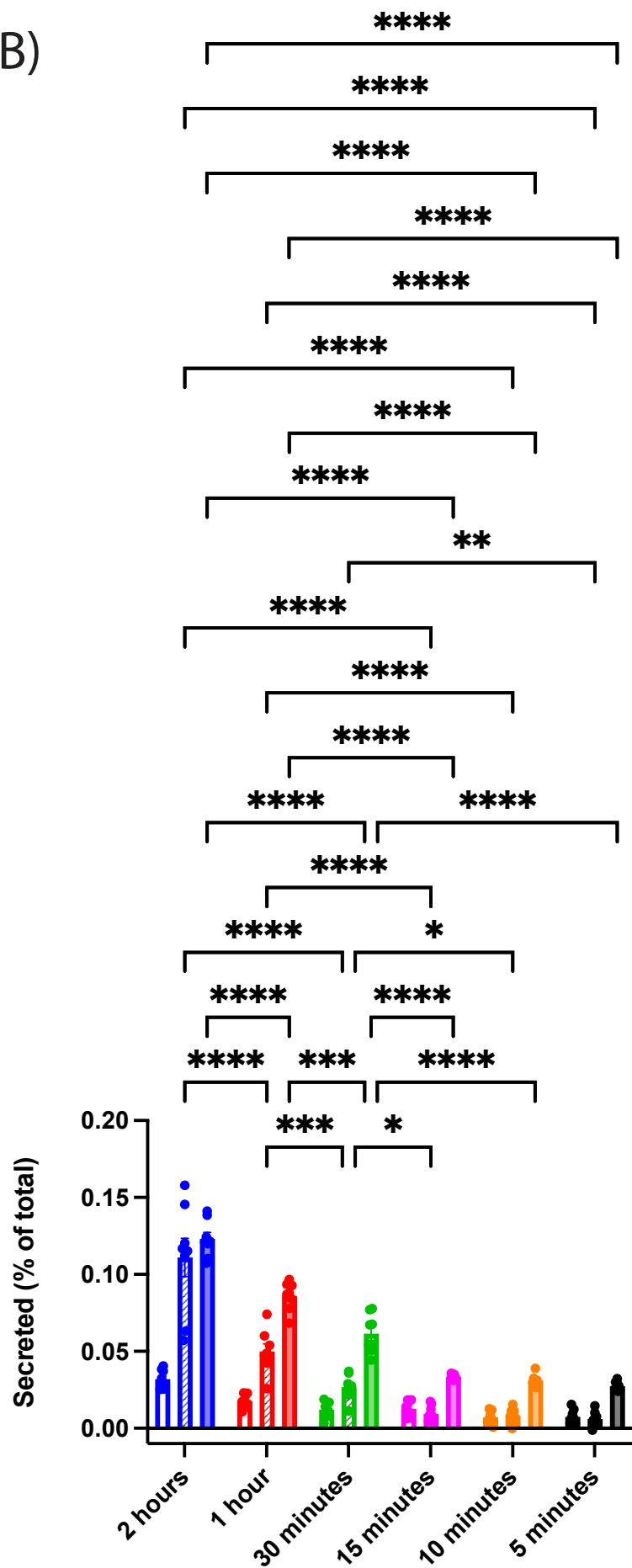

Supplement: Supplementary file 6 [file DataSheet6.pdf]

A)

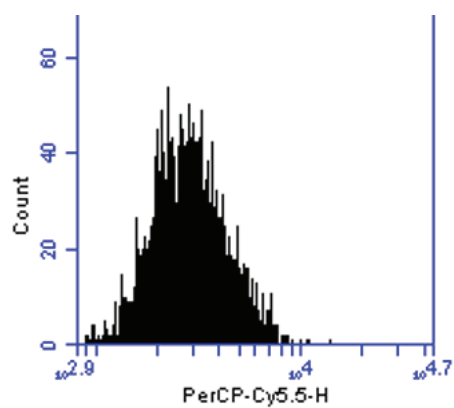

Untransfected

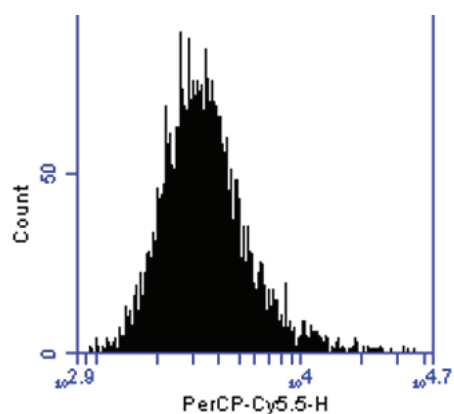

NPY-mCherry

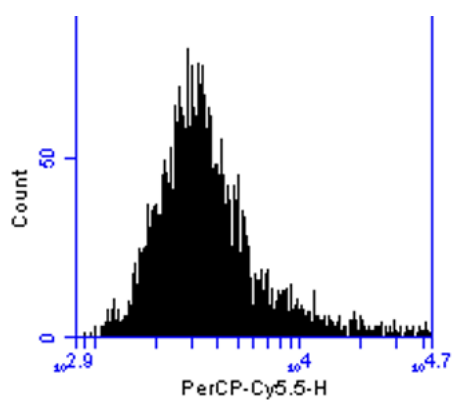

NPY-sfCherry2

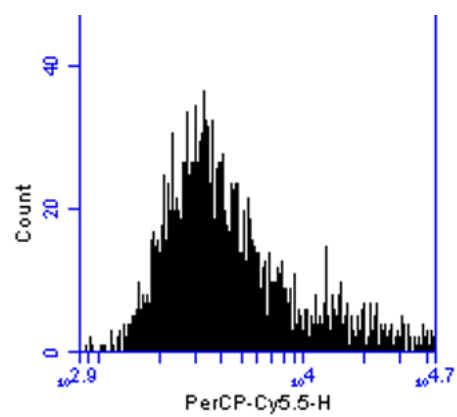

NPY-sfCherry3c

Supplement: Supplementary file 7 [file DataSheet7.pdf]
