# Supplementary material for: An optimized fluorescent reporter enables rapid and cost-effective quantification of regulated secretion from neuroendocrine cells
Source: Front Endocrinol (Lausanne). 2025 Aug 18;16:1640601. doi: 10.3389/fendo.2025.1640601 (PMC12400516; doi:10.3389/fendo.2025.1640601)
Supplement: Supplementary Table 1 — Sequence of gene blocks used to clone NPY and the respective indicated fluorescent reporters. [file Table1.pdf]

Table S1

NPY-mCherry	ATGTTAGGTAACAAGCGACTGGGGCTGTCCGGACTGACCCTCGCCCTGTCCCTGCTCGTGTGCCTGGGTGCGCTGGCCGAGGCGTACCC<br>CTCCAAGCCGGACAACCCGGGCGAGGACGCACCAGCGGAGGACATGGCCAGATACTACTCGGCGCTGCGACACTACATCAACCTCATC<br>ACCAGGCAGAGATATGGAAAACGATCCAGCCCAGAGACACTGATTTCAGACCTCTTGATGAGAGAAAGCACAGAAAATGTTCCCAGAA<br>CTCGGCTTGAAGACCCTGCAATGTGGGTGAGCAAGGGCGAGGAGGATAACATGGCCATCATCAAGGAGTTCATGCGCTTCAAGGTGCA<br>CATGGAGGGCTCCGTGAACGGCCACGAGTTCGAGATCGAGGGCGAGGGCGAGGGCCGCCCCTACGAGGGCACCCAGACCGCCAAGCT<br>GAAGGTGACCAAGGGTGGCCCCCTGCCCTTCGCCTGGGACATCCTGTCCCCTCAGTTCATGTACGGCTCCAAGGCCTACGTGAAGCACC<br>CCGCCGACATCCCCGACTACTTGAAGCTGTCCTTCCCCGAGGGCTTCAAGTGGGAGCGCGTGATGAACTTCGAGGACGGCGGCGTGGT<br>GACCGTGACCCAGGACTCCTCCCTGCAGGACGGCGAGTTCATCTACAAGGTGAAGCTGCGCGGCACCAACTTCCCCTCCGACGGCCCC<br>GTAATGCAGAAGAAGACCATGGGCTGGGAGGCCTCCTCCGAGCGGATGTACCCCGAGGACGGCGCCCTGAAGGGCGAGATCAAGCAG<br>AGGCTGAAGCTGAAGGACGGCGGCCACTACGACGCTGAGGTCAAGACCACCTACAAGGCCAAGAAGCCCGTGCAGCTGCCCGGCGCC<br>TACAACGTCAACATCAAGTTGGACATCACCTCCCACAACGAGGACTACACCATCGTGGAACAGTACGAACGCGCCGAGGGCCGCCACT<br>CCACCGGCGGCATGGACGAGCTGTACAAG
NPY-sfCherry2	ATGTTAGGTAACAAGCGACTGGGGCTGTCCGGACTGACCCTCGCCCTGTCCCTGCTCGTGTGCCTGGGTGCGCTGGCCGAGGCGTACCC<br>CTCCAAGCCGGACAACCCGGGCGAGGACGCACCAGCGGAGGACATGGCCAGATACTACTCGGCGCTGCGACACTACATCAACCTCATC<br>ACCAGGCAGAGATATGGAAAACGATCCAGCCCAGAGACACTGATTTCAGACCTCTTGATGAGAGAAAGCACAGAAAATGTTCCCAGAA<br>CTCGGCTTGAAGACCCTGCAATGTGGAAGCTTGAGGAGGACAACATGGCCATCATCAAGGAGTTCATGAGATTCAAGGTGCACATGGA<br>GGGCAGCGTGAACGGCCACGAGTTCGAGATCGAGGGCGAGGGCGAGGGCCACCCCTACGAGGGCACCCAGACCGCCAAGCTGAAGG<br>TGACCAAGGGCGGCCCCCTGCCCTTCGCCTGGGACATCCTGAGCCCCCAGTTCATGTACGGCAGCAAGGCCTACGTGAAGCACCCCGC<br>CGACATCCCCGACTACCTGAAGCTGAGCTTCCCCGAGGGCTTCACCTGGGAGAGAGTGATGAACTTCGAGGACGGCGGCGTGGTGACC<br>GTGACCCAGGACAGCAGCCTGCAGGACGGCCAGTTCATCTACAAGGTGAAGCTGCTGGGCATCAACTTCCCCAGCGACGGCCCCGTGA<br>TGCAGAAGAAGACCATGGGCTGGGAGGCCAGCACCGAGAGAATGTACCCCGAGGACGGCGCCCTGAAGGGCGAGATCAACCAGAGAC<br>TGAAGCTGAAGGACGGCGGCCACTACGACGCCGAGGTGAAGACCACCTACAAGGCCAAGAAGCCCGTGCAGCTGCCCGGCGCCTACA<br>ACGTGGACATCAAGCTGGACATCACCAGCCACAACGAGGACTACACCATCGTGGAGCAGTACGAGAGAGCCGAGGCCAGACACAGCA<br>CCTGA
NPY-sfCherry3c	ATGTTAGGTAACAAGCGACTGGGGCTGTCCGGACTGACCCTCGCCCTGTCCCTGCTCGTGTGCCTGGGTGCGCTGGCCGAGGCGTACCC<br>CTCCAAGCCGGACAACCCGGGCGAGGACGCACCAGCGGAGGACATGGCCAGATACTACTCGGCGCTGCGACACTACATCAACCTCATC<br>ACCAGGCAGAGATATGGAAAACGATCCAGCCCAGAGACACTGATTTCAGACCTCTTGATGAGAGAAAGCACAGAAAATGTTCCCAGAA<br>CTCGGCTTGAAGACCCTGCAATGTGGAAGCTTGAGGAGGACAACATGGCCATCATCAAGGAGTTCATGAGATTCAAGGTGCACATGGA<br>GGGCAGCGTGAACGGCCACGAGTTCGAGATCGAGGGCGAGGGCGAGGGCCACCCCTACGAGGGCACCCAGACCGCCAGGCTGAAGG<br>TGACCAAGGGCGACCCCCTGCCCTTCGCCTGGGACATCCTGAGCCCCCAGTTCATGTACGGCAGCAAGGCCTACGTGAAGCACCCCGC<br>CGACATCCCCGACTACCTGAAGCTGAGCTTCCCCGAGGGCTTCACCTGGGAGAGAGTGATGAACTTCGAGGACGGCGGCGTGGTGGCC<br>GTGACCCAGGACAGCAGCCTGCAGGACGGCCAGTTCATCTACAAGGTGAAGCTGCTGGGCATCAACTTCCCCAGCGACGGCCCCGTGA<br>TGCAGAAGAAGACCATGGGCTGGGAGGCCAGCACCGAGAGAATGTACCCCGAGGACGGCGCCCTGAAGGGCGAGATCAACCAGAGAC<br>TGAAGCTGAAGGACGGCGGTCACTACGACGCCGAGGTGAAGACCACCTACAGGGCCAAGAAGCCCGTGCAGCTGCCCGGCGCCTACG<br>ACGTGGACATCAAGCTGGACATCACCAGCCACAACGAGGACTACACCATCGTGGAGCAGTACGAGAGAGCCGAGGCCAGACACAGCA<br>CCTGA
