# Supplementary material for: An optimized fluorescent reporter enables rapid and cost-effective quantification of regulated secretion from neuroendocrine cells
Source: Front Endocrinol (Lausanne). 2025 Aug 18;16:1640601. doi: 10.3389/fendo.2025.1640601 (PMC12400516; doi:10.3389/fendo.2025.1640601)
Supplement: Supplementary Table 2 — *Detailed cost breakdown for ELISA vs NPY secretion assay. As one ELISA kit costs $678.50 and has a max loading capacity of 36 samples (loaded in duplicates), each sample costs $18.85. In contrast, using the fluorescence plate reader-based secretion assay, the cost per sample comes down to $1.50 ($0.72 for the plate and $0.78 for the transfection), which represents a ~12.5x cost reduction per sample compared to ELISA. The 316 samples used for this study would have required a total of 9 ELISA plates, bringing the total cost to $6106.50. In contrast, the total cost actually incurred for this study by using this newly developed secretion assay is only ~$478. [file Table2.pdf]

Table S2

Cost breakdown for optimized ELISA plate (36 samples determined to be max number of samples able to load)	***ELISA***	***NPY***
ELISA Cost (1 kit)	$678.50	$0
Greiner 96-well black plate	$0	$25.76
Lipofectamine 2000 (1.5mL)	$0	$776.16
Lipofectamine 2000 (1.5µl, 1rxn)	$0	$0.78
Cost per sample *	$18.85	$1.50
Total Cost for this Study (316 samples)	$6106.50	$478.32
